# Effect of Particle Wettability and Particle Concentration on the Enzymatic Dehydration of *n*‐Octanaloxime in Pickering Emulsions

**DOI:** 10.1002/anie.202013171

**Published:** 2020-12-21

**Authors:** Ana Maria Bago Rodriguez, Lukas Schober, Alessa Hinzmann, Harald Gröger, Bernard P. Binks

**Affiliations:** ^1^ Department of Chemistry University of Hull Hull HU6 7RX UK; ^2^ Faculty of Chemistry Bielefeld University Universitätsstrasse 25 33615 Bielefeld Germany

**Keywords:** biphasic catalysis, enzyme catalysis, Pickering emulsions, silica particles

## Abstract

Pickering emulsion systems have emerged as platforms for the synthesis of organic molecules in biphasic biocatalysis. Herein, the catalytic performance was evaluated for biotransformation using whole cells exemplified for the dehydration of *n*‐octanaloxime to *n*‐octanenitrile catalysed by an aldoxime dehydratase (OxdB) overexpressed in *E. coli*. This study was carried out in Pickering emulsions stabilised solely with silica particles of different hydrophobicity. We correlate, for the first time, the properties of the emulsions with the conversion of the reaction, thus gaining an insight into the impact of the particle wettability and particle concentration. When comparing two emulsions of different type with similar stability and droplet diameter, the oil‐in‐water (o/w) system displayed a higher conversion than the water‐in‐oil (w/o) system, despite the conversion in both cases being higher than that in a “classic” two‐phase system. Furthermore, an increase in particle concentration prior to emulsification resulted in an increase of the interfacial area and hence a higher conversion.

## Graphical abstract

The dehydration of *n*‐octanaloxime to *n*‐octanenitrile catalysed by an aldoxime dehydratase (OxdB) overexpressed in *E. coli* is carried out in Pickering emulsions stabilised solely with silica particles of different hydrophobicity. The effects of particle wettability and particle concentration are evaluated with respect to the conversion of the reaction.

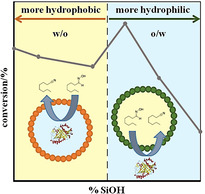

## Introduction

Enzymes are efficient biocatalysts that, due to their high chemo‐, regio‐ and stereo‐selectivity, play an important role in the synthesis of organic molecules under mild and sustainable conditions.[^1^, ^2^, ^3^] Enzymes are commonly active in water, while at the same time often organic substrates are poorly soluble in aqueous media. To increase the solubility of the substrate in the aqueous phase, a co‐solvent can be added. For very hydrophobic substrates a second organic phase, which acts as a reservoir for the substrates, can be utilised to convert higher amounts of material. For a sufficient mass transfer of the substrate from the organic phase into the aqueous phase for conversion by the enzyme, stirring is needed. However, mechanical stirring as well as long exposure to organic solvents often lead to enzyme deactivation. As a result, low conversions are often obtained from conventional biphasic systems.

To improve biphasic solvent systems for enzymatic transformations, co‐solvents,^[4]^ phase‐transfer reagents^[5]^ and thermoregulated biphasic systems^[6]^ have been explored. These methods, however, require the introduction of extra additives that can also be detrimental to the enzyme activity and often hamper downstream‐processing and product purification steps. Emulsions have emerged as an effective vehicle to carry out catalytic reactions as they drastically increase the interfacial area, compared to the biphasic system. Those stabilised by solid particles, so‐called Pickering emulsions,^[7]^ have gained particular attention as they show various advantages compared to other emulsions. Unlike surfactants, solid particles due to their high energy of detachment, may become irreversibly anchored at the interface of dispersed drops creating a steric barrier that prevents or inhibits coalescence.^[8]^ Moreover, as certain particles are biologically compatible and environmentally friendly, they are especially relevant in the field of biocatalysis as they might not inactivate the enzyme and simplify the separation and purification of the product. In some cases, the solid particles act simultaneously as the emulsifier and the catalyst[^9^, ^10^, ^11^, ^12^, ^13^, ^14^, ^15^, ^16^, ^17^, ^18^, ^19^, ^20^] while in other cases they are separate entities.[^20^, ^21^, ^22^, ^23^, ^24^, ^25^, ^26^, ^27^, ^28^, ^29^, ^30^, ^31^, ^32^, ^33^] The review by Pera‐Titus et al. refers to catalysis carried out in each of the previous systems as Pickering interfacial catalysis (PIC) and Pickering‐assisted catalysis (PAC), respectively.^[34]^ Examples of the use of Pickering emulsions to carry out both inorganic (with Pd, Rh, Al_2_O_3_ or Fe_2_O_3_)[^11^, ^15^, ^18^, ^22^, ^27^, ^30^] and biocatalytic[^9^, ^12^, ^13^, ^14^, ^17^, ^19^, ^20^, ^22^, ^23^, ^24^, ^25^, ^26^, ^29^, ^32^, ^33^] reactions have been extensively reported in the literature. Moreover, this strategy has been applied in continuous flow processes[^21^, ^22^, ^24^, ^29^, ^30^, ^31^] and cascade reactions,[^15^, ^19^, ^28^, ^31^] thus broadening the scope of applications.

**Scheme 1:**
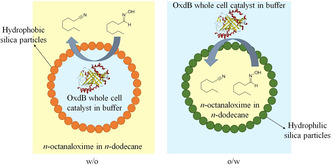
Biocatalytic transformation of *n*‐octanaloxime to *n*‐octanenitrile carried out in Pickering emulsions (w/o and o/w) stabilised by silica particles of different hydrophobicity. Although the starting material and the final product are slightly soluble in the aqueous phase, this has not been included for simplicity.

Aliphatic nitriles are attractive compounds from an industrial point of view as they are widely used as solvents and key intermediates for the large‐scale production of commodity chemicals such as surfactants as well as fine chemicals, agrochemicals and pharmaceuticals. Here we report the enzymatic dehydration of *n*‐octanaloxime to *n*‐octanenitrile in Pickering emulsions stabilised with silica particles of different wettability using *E. coli* cells containing the aldoxime dehydratase OxdB (Scheme 1). This process was chosen as it allows the formation of such desired aliphatic nitriles by means of an alternative, cyanide‐free enzymatic approach.[^4^, ^35^, ^36^, ^37^, ^38^] Besides the exciting properties of the enzyme for this purpose, the large‐scale availability of aldehydes (being, e.g., accessible by hydroformylation) as well as hydroxylamine (used for Beckmann rearrangement within nylon production on an industrial scale) is a further advantage which makes this biotransformation interesting also for technical purposes. OxdB is a heme protein that catalyses the dehydration of an aldoxime by formation of a nitrile through the coordination of the nitrogen atom of the aldoxime to the ferrous (Fe^2+^) heme.[^39^, ^40^] Despite this transformation already having been reported at high substrate loadings with high conversions and yields using co‐solvents,^[4]^ here we carry out the reaction in Pickering emulsions without adding further chemicals. In the Pickering emulsion system we suggest that the reaction takes place in the aqueous phase as the enzyme, even if it is confined in a cellular matrix, is highly sensitive to organic solvents. Hinzmann et al. reported conversions lower than 10 % for the same reaction with whole cells catalyst in pure organic media.^[41]^ Therefore, this implies first the diffusion of the substrate from the organic phase to the aqueous phase, followed by the diffusion of the product back to the organic phase.

We correlate the properties of the emulsions with the conversion of the reaction, thus gaining an insight into the impact of the effect of the particle wettability and particle concentration. Silica particles possessing various silanol contents (%SiOH) spanning from 100 % SiOH (most hydrophilic) to 15 % SiOH (most hydrophobic) were used. Despite the fact that the particle wettability can be tuned to stabilise the desired emulsion for the biphasic reaction, this parameter has not been addressed systematically so far. In fact, to the best of our knowledge, in the literature there is no such study that compares the conversion of a biocatalytic reaction in both emulsion types (o/w and w/o) with the same particle type by gradually changing the particle hydrophilicity. However, we acknowledge the work of various authors who addressed this topic. Zhang et al. prepared two particle types of different hydrophilicity by grafting methyl groups on the surface of hydrophilic TiO_2_ particles loaded with Ru.^[10]^ Each of these particle types stabilised a different emulsion type that was used to study the selective hydrogenation of benzene to cyclohexene. However, as the stability of each emulsion was not equivalent, the conversions of the reactions were not comparable. Meng et al. have recently prepared lipase‐immobilized mesoporous silica particles with grafted alkyl silanes of different chain lengths to obtain particles of various wettabilities that catalysed the hydrolysis of tributyrin to butyric acid.^[12]^ However, they were all partially hydrophobic and rendered w/o emulsions. Finally, Chen et al. coated cells with a porous calcium phosphate mineral shell and adsorbed various concentrations of a surfactant (sodium monododecyl phosphate) to obtain particles of different wettabilities.^[17]^ In this case, the particles were both the emulsifier and the catalyst and stabilised both emulsion types. However, due to the surface modification, the activity of the enzyme differed depending on the particle type used and the study of the conversion of the reaction was only conducted with one emulsion type.^[17]^

## Results and Discussion

*n*‐Octanaloxime and *n*‐octanenitrile are not surface‐active at the *n*‐dodecane‐water interface (Figure S1 and Figure S2, respectively) whereas *E. coli* cells can stabilise some oil droplets in water at a concentration higher than 0.025 wt. % (Figure S3–S5). *E. coli* cells contain both hydrophilic and hydrophobic moieties at the surface as the outer membrane is composed mainly of lipopolysaccharide, phospholipid and protein.^[42]^ As a result, it can be surface‐active at the oil‐water interface. In fact, Röllig et al. reported the stabilisation of emulsions with *E. coli* cells containing a lyase for the stereoselective carboligation of benzaldehyde to (*R*)‐benzoin.^[43]^ Herein, however, we prefer silica particles to be the sole emulsifier. Therefore, a low concentration of *E. coli* cells containing OxdB of 0.025 wt. % was selected to prepare emulsions.

**Figure 1:**
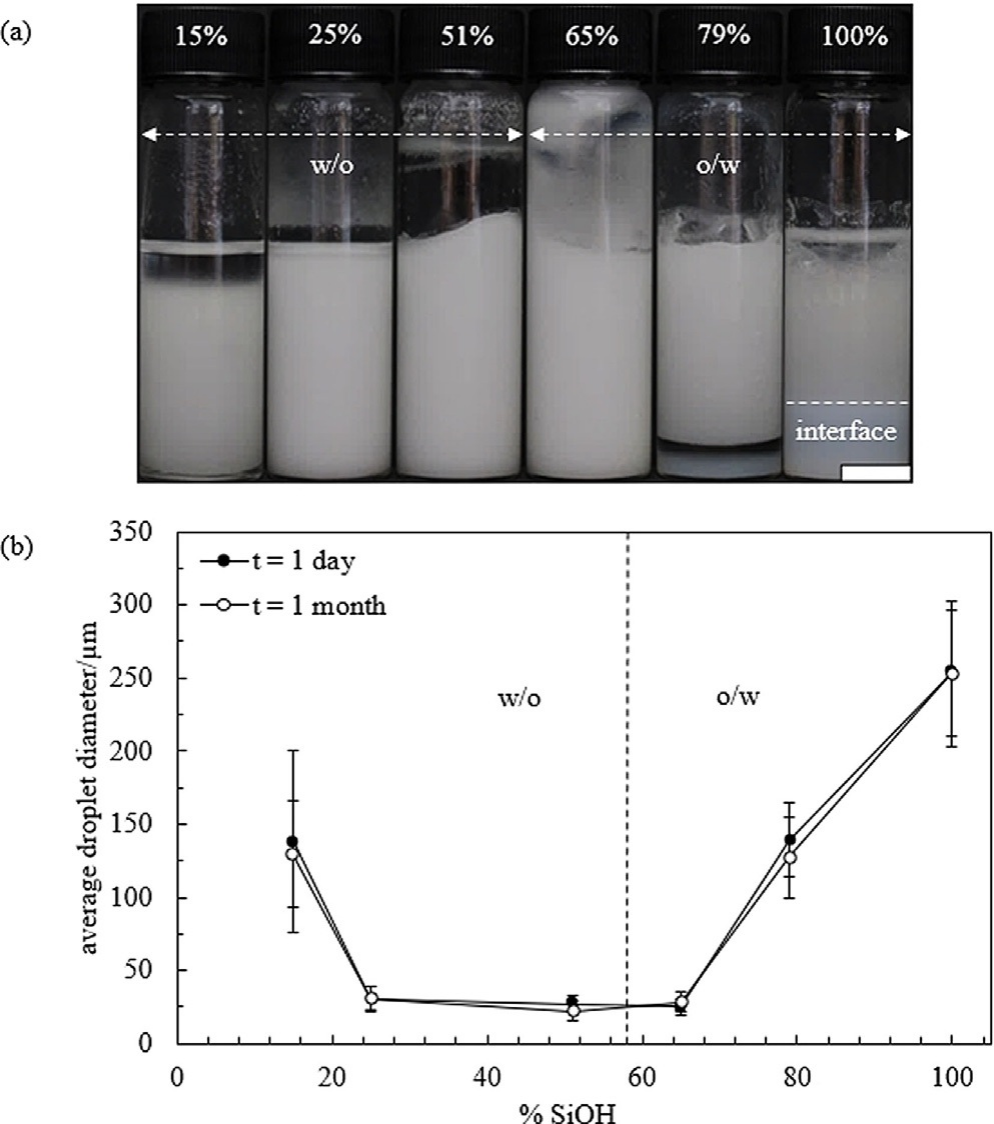
a) Appearance of emulsions prepared with silica particles of different hydrophobicity (% SiOH) 1 month after preparation. Each emulsion is obtained upon mixing equal volumes of an aqueous phase containing *E. coli* cells containing OxdB dispersed in 50 mM K_2_HPO_4_/KH_2_PO_4_ buffer (pH 7) and an organic phase containing n‐octanaloxime dissolved in *n*‐dodecane (10 mM). Silica particles with a SiOH content between 100 % and 65 % were dispersed in the aqueous dispersion while silica particles with a SiOH content between 51 % and 15 % were dispersed in the organic phase. The concentration of the different components in the emulsion are: 0.082 wt. % *n*‐octanaloxime, 0.025 wt. % *E. coli* cells containing OxdB and 1.14 wt. % silica particles. Scale bar=1 cm. b) Plot of the average droplet diameter after 1 day and 1 month versus SiOH content for emulsions in (a).

**Figure 2:**
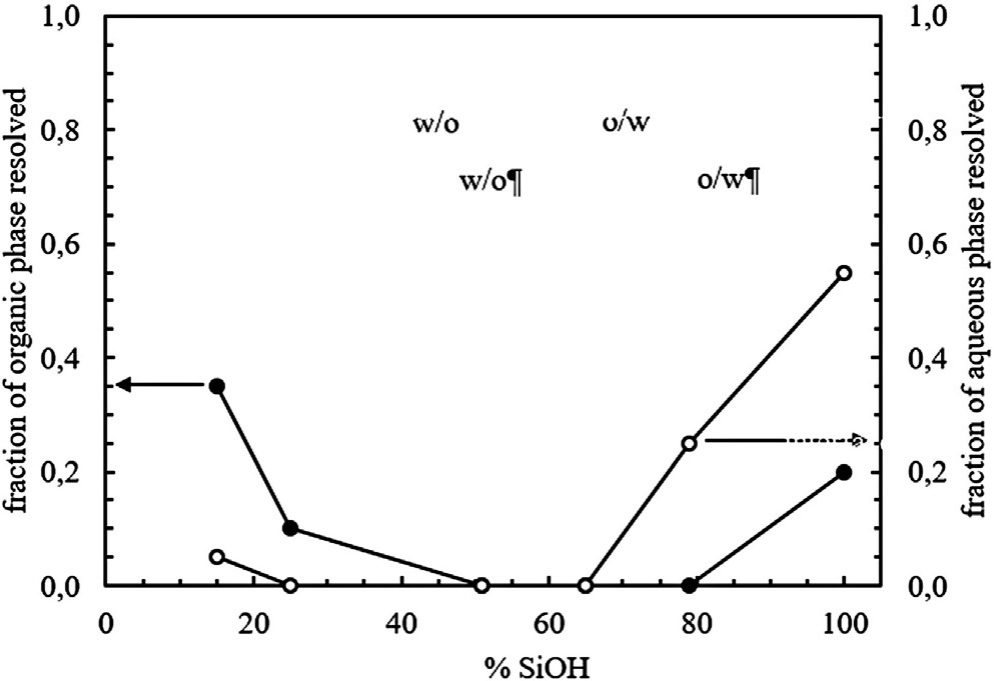
Plot of the fraction of organic and aqueous phase resolved after 1 month versus SiOH content for emulsions in Figure 1.

Emulsions were then prepared with silica particles of different hydrophobicity by keeping the concentration of *E. coli* cells containing OxdB and n‐octanaloxime in the emulsion constant (0.025 wt. % and 0.082 wt. %, respectively. We show in Figure S6 that emulsions containing *E. coli* cells behave the same with or without OxdB). The concentration of silica particles is 1.14 wt. % in all cases. Photos were taken one month after preparation (Figure 1 a) and the emulsion type was inferred from the drop test. Emulsions containing silica particles with a SiOH content from 100 % to 65 % were o/w emulsions whereas w/o emulsions were obtained from the most hydrophobic silica particles (SiOH content from 51 % to 15 %). In agreement with this transitional phase inversion, the average droplet diameter decreases upon approaching the inversion point (Figure 1 b and Figure S7) as shown by Binks and Lumsdon.^[44]^ The fraction of aqueous and organic phase resolved from emulsions was measured one month after preparation and they both also decrease approaching the point of phase inversion, in agreement with ref. [44] (Figure 2). The stability of the prepared emulsions, in terms of the average droplet diameter and the fraction of organic and aqueous phase resolved, remained unaltered for at least 1 month. The energy of detachment of particles from the oil‐water interface is several orders of magnitude higher than their thermal energy (*k* *T*) and so particles are thought to be irreversibly adsorbed at interfaces. Therefore, when creaming/sedimentation and coalescence are halted, emulsions remain unchanged for a long period of time. Hence no difference regarding the emulsion stability was observed between the emulsions after 48 h and 1 month.

**Figure 3:**
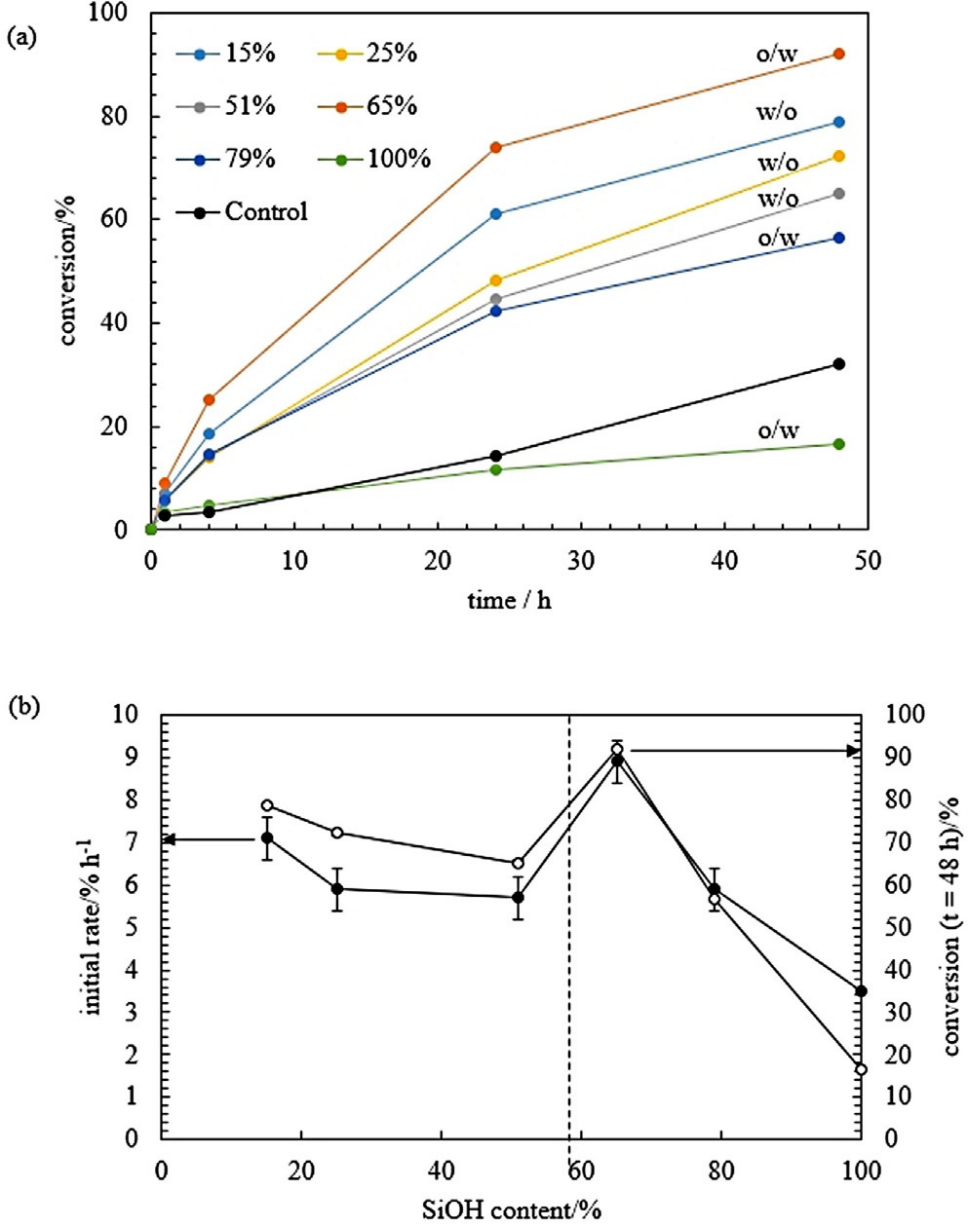
a) Plot of the conversion of the reaction of *n*‐octanaloxime into *n*‐octanenitrile versus time for the emulsions in Figure 1 and control. The control is the two‐phase system comprising of a 0.44 g L^−1^ *E*. *coli* cells (containing OxdB) dispersion in 50 mM K_2_HPO_4_/KH_2_PO_4_ buffer (pH 7) and a 10 mM solution of *n*‐octanaloxime in *n*‐dodecane. The emulsion type and % SiOH are given. b) Plot of the initial rate of the reaction (filled circles) and conversion of the reaction after 48 h (empty circles) versus SiOH content.

A second set of emulsions was prepared to study the conversion of the reaction therein. As described in the experimental section, samples from the emulsion cream were withdrawn at different times and the conversion of the reaction was measured by gas chromatography (GC). In all cases, the conversion increases with time but the reaction becomes slower at long time (Figure 3 a). In enzyme kinetics, *V*_max_ is the theoretical maximal turnover rate. *K*_m_ is defined as the substrate concentration where *V*_max_/2 is reached and depends on the type of enzyme and substrate. This means that even if the substrate concentration increases above approx. 2 *x* *K*_m_, an increase of the turnover rate will not occur anymore. The observed decrease in the rate with time in all our experiments could be due to either one or a combination of the following reasons: (i) deactivation of the enzyme (due to the solvent, temperature or time), (ii) insufficient diffusion of the substrate from the organic to the aqueous phase and (iii) low amount of substrate available (as the reaction proceeds, the amount of *n*‐octanaloxime might be too low so the condition of 2 *x* *K*_m_ can no longer be reached in the aqueous phase). As observed in Figure 3 a, the conversion in the Pickering emulsions is higher compared to the two‐phase control in all cases, apart from that obtained from the emulsion prepared with the most hydrophilic silica particles which gave a similar result. Due to the large volume of aqueous phase resolved, a high amount of *E. coli* cells containing OxdB might not be available for the reaction. In the control experiment, the aqueous phase containing the enzyme was homogenised for 2 min at 13 000 rpm and the two phases were left to stand undisturbed as the emulsions were also not stirred while the reaction was taking place. The conversion reached with other controls (with/without homogenisation of the aqueous phase and with/without stirring) is shown in Figure S8. Stirring enhances the diffusion of the components and high conversions are achieved as shown in Figure S8(a) by comparing the blue and grey points with the yellow and orange points. The conversions obtained with and without homogenisation of the aqueous dispersion containing the cells are similar both for the stirred (grey and blue points) and for the non‐stirred (yellow and orange points) case. Therefore, homogenisation does not seem to affect the activity of the enzyme. However, when the two phases are homogenised together the conversion is very low for both the stirred and non‐stirred case (Figure S8(b)). This suggests that the organic phase deactivates considerably the enzyme during homogenisation. It is noteworthy that this deactivation effect does not occur when the two phases are homogenised in the presence of silica particles as they protect the enzyme from the organic phase by forming a protective layer of particles at the oil‐water interface. *n*‐Octanaloxime is slightly soluble in water. Therefore, another control experiment was carried out in a monophasic system, where *n*‐octanaloxime was placed as a solid in an aqueous phase containing the enzyme (0.44 g L^−1^) and was stirred at 1000 rpm. The conversion after 1, 4 and 24 h was as low as 2.9, 5.7 and 9.7 %, respectively. All these controls highlight the advantages of carrying out the reaction in an emulsion system, in terms of achieving high conversions and protecting the enzyme from the organic solvent.

Furthermore, an initial rate was calculated as described in the ESI (section 1.2.5). As shown in Figure 3 (b, filled circles), the plot of the initial rate follows the same pattern as the plot of the conversion at *t*=48 h versus %SiOH (Figure 3 (b, empty circles)). The highest conversion is obtained with the most stable emulsion to creaming and coalescence that contains oil droplets with the smallest average droplet diameter (65 % SiOH). Then, the degree of conversion decreases either by increasing or decreasing the particle hydrophobicity. However, for the most hydrophobic silica particles, conversions are comparable. A surprising result is the high conversion obtained in the w/o emulsion stabilised with the most hydrophobic silica particles (15 % SiOH), compared to that measured from the w/o emulsion prepared with silica particles with 25 % SiOH groups. Despite the fraction of coalesced oil being higher and the average droplet diameter is about 10 times larger in the former emulsion, the conversion measured is almost 10 % higher. This could indicate that silica particles of different hydrophobicity might interact in a different way with the *E. coli* cells and this could finally influence the conversion of the reaction. The particle contact angle at the oil‐water interface varies depending on the particle hydrophobicity as shown in Figure S9(a) for two partially hydrophobic silica particles. By increasing the particle hydrophobicity (lower SiOH content) particles protrude more into the oil phase of the w/o Pickering emulsion. As a result, the fraction of the particle surface in oil is higher for more hydrophobic particles. This could explain the increase of the conversion detected in the w/o emulsions by increasing the particle hydrophobicity. *E. coli* cells containing the enzyme in the water droplets may be more protected from the organic environment with 15 % SiOH silica particles than with 25 % or 51 % SiOH silica particles. The average droplet diameter in this case would not be the main factor governing the extent of the conversion as the diffusion of organic molecules across the interface is expected to be high. This is not the case for the o/w emulsions as creaming is observed in some cases. As a result, some *E. coli* cells containing the enzyme dispersed in the aqueous phase released will not be accessible for reaction.

Forty‐eight hours after emulsion preparation, emulsions were broken by centrifugation, recovering the two incompatible phases together with a thin layer of emulsion cream that could not be broken completely. The conversion of the reaction was measured in both phases by GC. The conversions measured in the organic phases separated from the emulsions prepared with silica particles of different hydrophobicity are shown in Figure S9(b) (empty circles). This is referred to as the work‐up of the organic phase (WU OP). The conversion of the reaction was also measured in the aqueous phase as both *n*‐octanaloxime and *n*‐octanenitrile are slightly soluble in water (calculated values using Advanced Chemistry Development Software V11.02 reported in SciFinder: Octanaloxime: log *P*=2.927; Octanenitrile: log *P*=2.723). Similar conversions were measured in the aqueous phase which could imply that the partitioning of the two molecules between the organic and the aqueous phases is similar. As expected, the areas corresponding to both molecules in the aqueous phase were very low compared to the ones measured in the organic phase so they have been neglected for the analysis. The conversion of the reaction in the emulsion cream after 48 h (filled circles) is compared with that obtained from the organic phase separated by centrifugation (empty circles) after the same period of time (Figure S9(b)). In general, the two values are in good agreement and they match completely for the emulsion prepared with silica particles of intermediate hydrophobicity (no aqueous or organic phase resolved).

As mentioned in the introduction, this is the first time that the conversion of a biocatalytic reaction is followed in Pickering emulsions prepared with the same particle type (silica) by progressively increasing the particle hydrophobicity. One of the reasons that could explain the absence in the literature of a systematic study could be that it is difficult to prepare two emulsions differing in type with a similar stability and average droplet diameter. Zhang et al. prepared two emulsion types with similar droplet diameters (ca. 60 μm) but different stabilities to study the hydrogenation of benzene to cyclohexene.^[10]^ Despite the o/w emulsion registering a slightly higher conversion, selectivity and yield compared to the biphasic system, compared with the w/o emulsion the values are within the same range. Meng et al. prepared lipase‐immobilized mesoporous silica particles with grafted alkyl silanes of different chain lengths.^[12]^ The three‐phase contact angle increased by increasing the silane carbon chain length, but the particles were all slightly hydrophobic with contact angles between 95° and 145°. As in our case, emulsions prepared with particles of intermediate hydrophobicity with a contact angle close to 90° display the highest emulsion stability with the lowest average droplet diameter and in these cases the highest conversion was measured.^[12]^ Unlike the previous examples, due to the availability of a wide range of silica particles of different wettability, here the emulsion stability can be tuned to a specific extent in order to fulfil all the requirements. Emulsions stabilised with silica particles with 51 % and 65 % SiOH groups have similar stabilities (no aqueous or organic phase resolved) and average droplet diameters (≈25 μm) but render a w/o and o/w emulsion, respectively. The conversion of the reaction after 48 h is considerably different in each case, being 65 % and 92 % for the w/o and o/w emulsion, respectively. The reason for this remarkable difference is still under investigation. As pointed out before, it could be related to the different interaction of *E. coli* cells with the silica particles but it could also indicate that the confinement of the biocatalyst in water droplets of a w/o emulsion could reduce the conversion compared to the o/w system where the biocatalyst diffuses freely through the continuous phase. Moreover, as pointed out previously in the w/o emulsion prepared with 51 % SiOH silica particles, the particles protrude more into the organic phase. On the contrary, in the o/w emulsion prepared with the 65 % SiOH particles, particles are slightly more wetted by the aqueous phase. Therefore, one would expect less deactivation of the enzyme in the o/w emulsion as the interaction of the enzyme with the organic solvent is slightly lower and hence a higher conversion can be achieved. However, none of these hypotheses has been proven yet.

**Figure 4:**
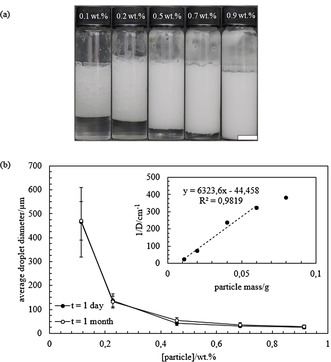
a) Appearance of o/w emulsions prepared at different concentrations of 65 % SiOH silica particles (with respect to emulsion) 1 month after preparation. Each emulsion is made by mixing equal volumes of an aqueous phase containing *E. coli* cells containing OxdB and silica particles dispersed in 50 mM K_2_HPO_4_/KH_2_PO_4_ buffer (pH 7) and an organic phase containing *n*‐octanaloxime dissolved in n‐dodecane (10 mM). The concentration of the different components in the emulsion are: 0.082 wt. % *n*‐octanaloxime and 0.025 wt. % *E. coli* cells containing OxdB. Scale bar=1 cm. b) Plot of the average droplet diameter versus particle concentration in the emulsions in (a) 1 day and 1 month after preparation. Inset: inverse of average droplet diameter as a function of the mass of 65 % SiOH silica particles in the aqueous dispersion.

**Figure 5:**
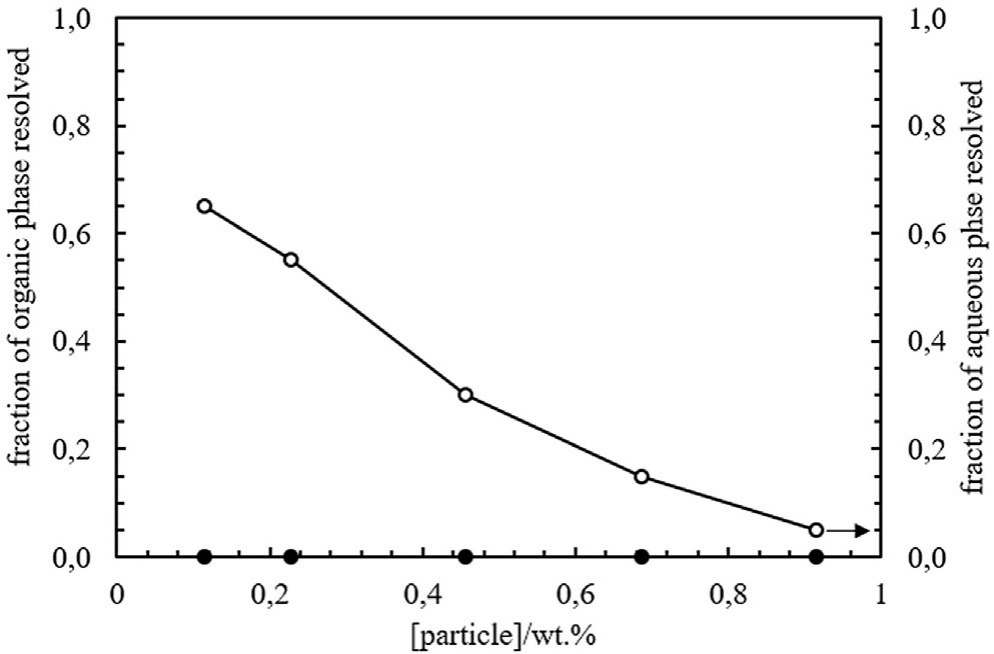
Fraction of aqueous (empty circles) and organic (filled circles) phase resolved from the o/w emulsions in Figure 4 a prepared with different concentrations of 65 % SiOH silica particles 1 month after preparation.

The influence of an increase of the interfacial area on the conversion of the reaction carried out in Pickering emulsions was evaluated. Oil‐in‐water emulsions at a fixed concentration of substrate and *E. coli* cells containing OxdB and different silica particle concentrations (0.1, 0.2, 0.5, 0.7 and 0.9 wt.%) were prepared with 65 % SiOH silica particles (Figure 4 a). From visual inspection after one month, the extent of creaming decreases by increasing the particle concentration and coalescence is fully inhibited in all cases (Figure 5). Optical microscope images were taken one day and one month after preparation (Figure S10). The average droplet diameter decreases by increasing the concentration of particles following the limited coalescence model of particle‐stabilised emulsions (Figure 4 b). This analysis is described in the ESI (section 2.3).

**Figure 6:**
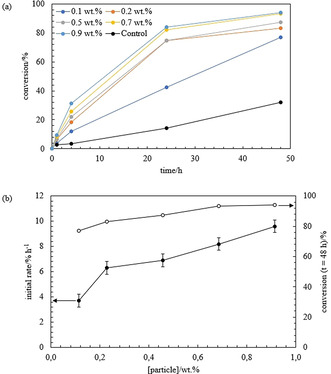
a) Plot of the conversion of the reaction of *n*‐octanaloxime into *n*‐octanenitrile versus time for the o/w emulsions in Figure 4 a prepared with different concentrations of 65 % SiOH silica particles and control. The control is the two‐phase system comprising of a 0.44 g L^−1^ *E. coli* cells (containing OxdB) dispersion in 50 mM K_2_HPO_4_/KH_2_PO_4_ buffer (pH 7) and a 10 mM solution of n‐octanaloxime in *n*‐dodecane. b) Plot of the initial rate of the reaction (filled circles) and conversion of the reaction after 48 h (empty circles) versus particle concentration in the emulsion.

Samples from the emulsion cream were taken at different times and the conversion of the reaction was measured by GC. As shown in Figure 6 a, the conversion increases with time for all the emulsions stabilised with 65 % SiOH silica particles at different concentrations and the values are higher compared with the control (two‐phase system). Moreover, at any specific time, the degree of conversion increases by increasing the particle concentration. The conversion of the reaction measured from the emulsion prepared with 0.9 wt. % of particles after 48 h is of the same order as the one obtained with 1.14 wt. %. This is explained by the fact that both emulsions have the same average droplet diameter and they are stable to creaming and coalescence. At high particle concentration the average diameter does not decrease further, in agreement with the limited coalescence model. This behaviour was also reported by Meng et al.,^[21]^ Sun et al.^[19]^ and Zhang and co‐workers.^[29]^ In ref. [29], though, a reduction of the catalytic efficiency was reported at high particle concentration due to a high particle coverage that hindered the encounter of the reactants (in oil) with the catalyst (in water). Zhang et al. reported the impact of the emulsion droplet diameter in the hydrogenation of benzene by varying the stirring rate during emulsification.^[10]^ The conversion, selectivity and yield increased by decreasing the size of the oil droplets.

An initial reaction rate was calculated as in the previous section and it increases with particle concentration (filled circles), following the same pattern as the plot for the conversion in the emulsion after *t*=48 h (empty circles) (Figure 6 b). After 48 h, emulsions were separated by centrifugation to recover the two phases and the conversion of the reaction in each phase was measured by GC. The conversions in the organic phase after work‐up as well as the conversions in the emulsion cream after 48 h are included in Figure S11 and they are in agreement, except for the emulsion prepared with 0.1 wt. % silica particles. Again, the conversions measured from the aqueous phase are similar to those obtained from the organic phase, which indicates a similar partitioning of both molecules between the two phases. Moreover, the areas measured in the aqueous phase are considered negligible compared to those measured from the organic phase.

**Figure 7:**
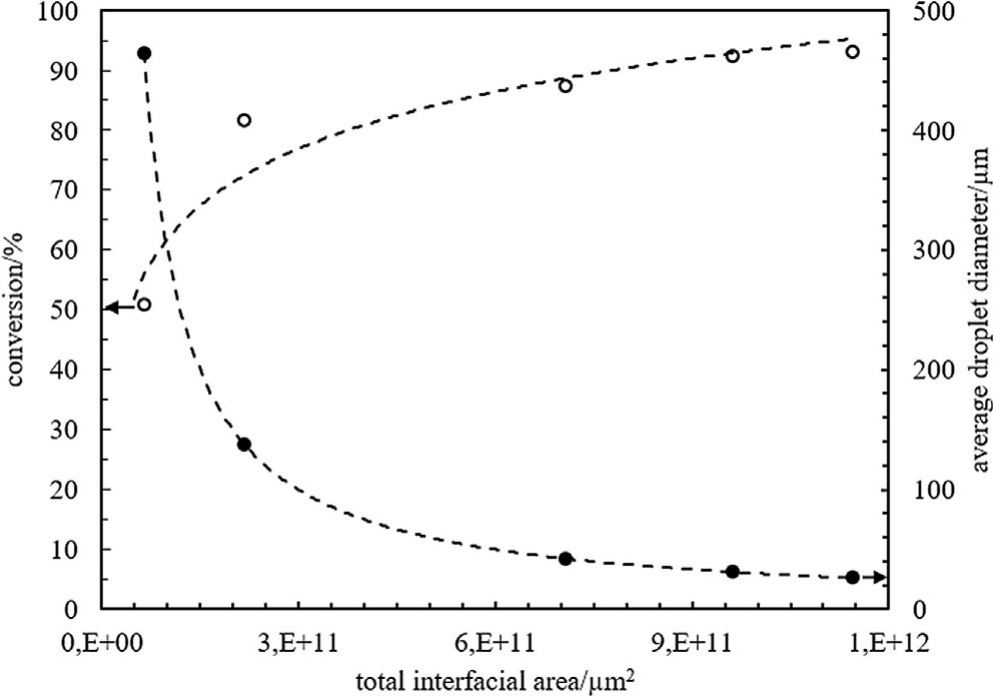
Plot of the conversion of the reaction in the organic phase recovered after centrifugation (empty circles) and average droplet diameter (filled circles) versus the total interfacial area in the o/w emulsions prepared with different concentrations of 65 % SiOH silica particles (Figure 4 a). The dotted lines are a guide for the eye.

Finally, the total interfacial area in emulsions prepared at different concentrations of 65 % SiOH silica particles was calculated as described in the ESI. Figure 7 shows the conversion of the reaction against the total interfacial area in the emulsion and the average droplet diameter and confirms that the increase in the conversion is due to the increase in the total interfacial area. The low conversions measured from emulsions prepared at low particle loadings could also be related to the fact that some amount of the biocatalyst is not available for the reaction as it remains in the resolved aqueous phase after creaming.

Another important advantage of carrying out the reaction in Pickering emulsions is that once the reaction is completed, the two phases can be separated by centrifugation. Thus, this elegant type of phase separation also enables an efficient recycling of the aqueous phase containing the biocatalyst (which often is difficult or even impossible when “standard emulsions” or “classic” two‐phase systems are used in biotransformations). The organic phase containing the product can be removed easily and a fresh organic phase containing *n*‐octanaloxime can be added to carry out the next cycle after homogenisation. The recyclability study was evaluated for 3 subsequent cycles in an emulsion stabilised with partially hydrophobic silica particles (65 % SiOH) and the conversion of the reaction measured after 1 hour is ≈6.5 % for all the cycles as shown in Table S5. The appearance of the emulsion and the average droplet diameter do not change significantly after each cycle. Hence this system can be recycled several times with no significant loss of activity, highlighting the positive effect of silica particles to protect the enzyme from the organic solvent.

## Conclusion

Pickering emulsions have been demonstrated to be a promising tool to perform catalytic reactions, thus representing an attractive alternative to the typical biphasic system. Here we reported the enzymatic dehydration of *n*‐octanaloxime to *n*‐octanenitrile in Pickering emulsions stabilised with silica particles of different hydrophobicity using *E. coli* cells with OxdB enzyme. To the best of our knowledge, this is the first time the conversion of a reaction is followed systematically in emulsions stabilised by the same particle type but of increasing hydrophobicity. The highest conversion after 48 h was accomplished in an o/w Pickering emulsion stabilised with silica particles of intermediate wettability. This precise control in tuning the particle hydrophobicity enabled the preparation of two kinds of emulsion of different type but with similar stability and droplet diameter. By comparing the conversion of the reaction in these emulsions, it appears to be higher in an o/w (92 %) than in a w/o (65 %) emulsion. However, in both cases the conversion was higher than that measured from a control without homogenisation. Finally, by increasing the particle concentration upon the preparation of the Pickering emulsions, emulsions with smaller average droplet diameters were prepared. The consequent increase in the total interfacial area resulted in an increase in conversion. Furthermore, in contrast to many traditional emulsions or two‐phase systems used in biotransformations, this Pickering emulsion system could be recycled several times without significant loss of enzyme activity, proving the positive effect of silica particles in protecting the enzyme from the organic solvent.

## Conflict of interest

The authors declare no conflict of interest.

## Supporting information

As a service to our authors and readers, this journal provides supporting information supplied by the authors. Such materials are peer reviewed and may be re‐organized for online delivery, but are not copy‐edited or typeset. Technical support issues arising from supporting information (other than missing files) should be addressed to the authors.

Supplementary
