# Supplementary material for: Effect of Particle Wettability and Particle Concentration on the Enzymatic Dehydration of n‐Octanaloxime in Pickering Emulsions
Source: Angew Chem Int Ed Engl. 2020 Dec 21;60(3):1450–7. doi: 10.1002/anie.202013171 (PMC7839585; doi:10.1002/anie.202013171)

Angewandte Chemie
Eine Zeitschrift der Gesellschaft Deutscher Chemiker

Supporting Information

Effect of Particle Wettability and Particle Concentration on the Enzymatic Dehydration of *n*-Octanaloxime in Pickering Emulsions

Ana Maria Bago Rodriguez, Lukas Schober, Alessa Hinzmann, Harald Gröger, and Bernard P. Binks**

anie_202013171_sm_miscellaneous_information.pdf

ESI

Table of contents

1. Experimental 2

1.1 Materials 2

1.2 Methods 2

1.2.1 Oxd expression in E. coli 2

1.2.2 Activity test with OxdB 3

1.2.3 Synthesis of n-octanaloxime 3

1.2.4 Preparation and characterisation of emulsions 4

1.2.5 Determination of the conversion of the reaction 6

1.2.6 Calculation of the total interfacial area in emulsions 8

1.2.7 Recyclability study 8

2. Results and Discussion 8

2.1 Controls to check the surface-activity of the substrate and the product 8

2.2 Effect of the concentration of *E. coli* cells on emulsion stability 9

2.3 Limited coalescence model of particle-stabilised emulsions 10

3. References 11

4. Appendix 12

1. Experimental

1.1 Materials

For the OxdB expression in *E. Coli* BL21-CodonPlus(DE3)-RIL cells (Fisher Scientific) were used. Luria broth (LB)-medium, terrific borth (TB) medium, Carbenicillin, Chloramphenicol, D-lactose and D-glucose were purchased from Carl Roth and agar-agar and the salts for the preparation of the potassium phosphate buffer (K_2HPO_4/KH_2PO_4) were purchased from VWR.

n-Octanal was purchased from Sigma Aldrich and was used without further purification. Chemicals for oxime synthesis were sodium carbonate (Carl Roth), ethanol (VWR), hydroxylamine hydrochloride (Alfa Aesar) and *n*-hexane (VWR) and were used as received. *n*-Octanenitrile was used as a reference compound for gas chromatography (GC) analysis and was purchased from TCI Chemicals.

For the preparation of emulsions, *n*-dodecane (> 99%) was purchased from Alfa Aesar and was passed twice through a basic alumina column to remove polar impurities. Water was purified through a RiOs-DI 3 UV water purification system (Merck Millipore) comprising of an ion exchange cartridge and a UV lamp. Fumed silica particles were kindly supplied by Wacker-Chemie and were used as received. Particles of different SiOH content (100%, 79%, 65%, 51%, 25% and 15%) were used. The primary particles are approximately spherical of diameter between 5 and 30 nm although they can aggregate into larger units of about 200 nm in diameter. Hydrophilic silica was silylated to various extents by reaction with dichlorodimethylsilane in the presence of water followed by drying at 300 °C for 2 h, leaving the particle surfaces containing silanol (SiOH) and dimethylsilane ($SiOSi(CH_3)_2$) groups.[1] The silanol content was determined by titration with aqueous NaOH, while the carbon content was measured using a carbon analyzer. From a previous investigation *via* the immersion test,[2] it is clear that the silanol content is proportional to particle wettability and we used the former to describe it.

Ethyl acetate (EtOAc) (p.a., Fisher Chemicals) was used as received to halt the reaction under study.

1.2 Methods

1.2.1 Oxd expression in E. coli

E. coli BL21-CodonPlus(DE3)-RIL cells harboring the plasmids with the OxdB-gene (Table S1) were stored as glycerol stocks at -80 °C. To prepare the pre-culture, a sample from the

glycerol stock was plated on 5 mL of Luria broth (LB)-agar containing 100 μg mL^{-1} carbenicillin and 34 μg mL^{-1} chloramphenicol (used as antibiotic) and it was incubated for 12 – 18 h at 37 °C under stirring at 180 rpm. Main cultures for the expression of OxdB were performed using TB-autoinduction medium. Sterile 20 g L^{-1} lactose solution in Milli-Q water (160 mL) and sterile 50 g L^{-1} D-glucose solution in Milli-Q water (16 mL) were added to 1,424 mL of sterile TB-medium (Carl Roth) in a 2 L Erlenmeyer flask. 100 μg mL^{-1} carbenicillin and 34 μg mL^{-1} chloramphenicol were added to the medium. Main cultures were inoculated with 1% (16 mL) of the OxdB pre-culture and incubated for 2 h at 37 °C and 150 rpm. Afterwards, OxdB-cultures were cultivated at 30 °C for 72 h and 150 rpm.

Cell harvest was performed at 4,000 g for 15 min and 4 °C (Thermo Scientific Heraeus multifuge X3R). The supernatant was discarded and cells were washed three times with 50 mM K_2HPO_4/KH_2PO_4 buffer at pH 7.0. The biomass was determined (bio wet weight (bww)) and cells were re-suspended in 50 mM K_2HPO_4/KH_2PO_4 buffer (pH 7.0) to a final concentration of 333 mg mL^{-1} cells in buffer. Cell suspensions were stored in a fridge at 4 °C or on ice before use in biotransformations.

1.2.2 Activity test with OxdB

Standard activity assays of Oxds in whole cells using *n*-octanaloxime were performed using whole cells (bww) (33 mg/mL or 0.44 mg/mL) in a total volume of 0.5 mL in 1.5 mL micro-reaction tubes with 100 or 10 mM oxime concentration. The reaction was conducted in 50 mM K_2HPO_4/KH_2PO_4 buffer (pH 7.0) containing 10% (v/v) ethanol as co-solvent. The activity assay was performed at 30 °C and 1,400 rpm in an Eppendorf ThermoMixer. The reaction was stopped by addition of 500 μL EtOAc and extraction of substrates and products into the organic phase. Phase separation was enhanced by pulse centrifugation to 14,000 g at room temperature. The organic phase was analyzed by GC (Table S2).

1.2.3 Synthesis of n-octanaloxime

Sodium carbonate (0.75 eq) was dissolved in distilled water with 5% (v/v) of ethanol. Afterwards hydroxylamine hydrochloride (1.5 eq) was added to the stirred solution. Octanal (1 eq) was then added dropwise. The reaction mixture was stirred at room temperature for 12 h, during which the oximes precipitated as colourless solids. The reaction progress was monitored by thin-layer chromatography (TLC). After completion of the reaction, the crude product was isolated as a colourless solid by filtration. The crude product was rinsed with

distilled water (100 mL) before drying *in vacuo*. Pure *n*-octanaloxime was obtained after recrystallization from *n*-hexane.

NMR spectra were recorded on a Bruker Avance III 500 at a frequency of 500 MHz (^{1}H) or 125 MHz (^{13}C). The chemical shift δ is given in ppm and referenced to the corresponding solvent signal ($CDCl_3$). They are given in the Appendix.

Accurate mass nano-electrospray ionization (ESI) measurements were performed using a Q-IMS-TOF mass spectrometer Synapt G2Si (Waters GmbH, Manchester, UK) in resolution mode, interfaced to a nano-ESI ion source. Nitrogen served both as the nebulizer gas and as the dry gas for nano-ESI. Nitrogen was generated by a nitrogen generator NGM 11. Helium 5.0 was used as buffer gas in the ion-mobility spectrometry (IMS) entry cell and nitrogen 5.0 was used for IMS separations. 1,3-Dicyanobenzene is used as electron transfer reagent in electron-transfer dissociation (ETD). Samples were dissolved in acetonitrile and introduced by static nano-ESI using in-house pulled glass emitters.

Yield: 67%; ^{1}H-NMR (500 MHz, $CDCl_3$): *Z*-**5**: δ 7.41 (t, *J* = 6.1 Hz, 1H), 2.18 (td, *J* = 7.5, 6.2 Hz, 2H), 1.48 (m, 2H), 1.31 (m, 8H), 0.87 (m, 3H), *E*-**5**: δ 6.71 (t, *J* = 5.5 Hz, 1H), 2.37 (td, *J* = 7.6, 5.5 Hz, 2H), 1.48 (m, 2H), 1.31 (m, 8H), 0.87 (m, 3H); ^{13}C-NMR (125 MHz, $CDCl_3$): *Z*-**5**: δ 152.45, 31.36, 25.84, 29.57, 22.48, 14.06. *E*-**5**: δ 153.05, 31.66, 26. 35, 25.08, 22.48, 14.05. IR (neat): 1669.7 cm^{-1}; HRMS (ESI, positive ions) $[C_8H_{17}NOH]^+$ *m/z* (calcd) = 144.13829, *m/z* (found) = 144.1383.

1.2.4 Preparation and characterisation of emulsions

(a) Controls to check the surface-activity of the substrate and the product

Emulsions with an aqueous phase consisting of a 50 mM K_2HPO_4/KH_2PO_4 buffer (pH = 7) and an organic phase containing either *n*-octanaloxime or *n*-octanenitrile in *n*-dodecane were homogenised with an Ultra-turrax homogeniser (IKA T25 digital) with a dispersing element of 8 mm (stator diameter). Mixing was kept at 13,000 rpm for 2 min. The concentration of *n*-octanaloxime varied from 0.01 to 0.5 M while the concentration range studied for *n*-octanenitrile was 0.01 to 1 M. Photos of the vials were taken with a digital camera (Canon EOS 1100D) at different times to assess the emulsion stability.

(b) Effect of the concentration of *E. coli* cells on emulsion stability

Emulsions of a 10 mM *n*-octanaloxime solution in *n*-dodecane and a *E. coli* cells solution in 50 mM K_2HPO_4/KH_2PO_4 buffer (pH = 7) were prepared as stated above. The concentration of *E. coli* cells which will be used in the emulsions prepared with silica particles will be selected from this experiment and will be that so *E. coli* cells are surface-inactive. Therefore, we will ensure that emulsion stability is solely given by the silica particles. The concentration range of *E. coli* cells studied is shown in Table S3. For clarity, concentrations are given both in the aqueous dispersion and in the emulsion. The concentration of cells in the primary solution is approximately 330 g L^{-1}. Therefore, a 33 g L^{-1} stock solution was used to prepare the above dispersions.

Photos of the emulsions were taken at different times with a digital camera (Canon EOS 1100D). The emulsion type was inferred from the drop test. This consists in determining whether an emulsion drop disperses in the aqueous phase (o/w emulsion) or in the organic phase (w/o emulsion). Optical microscope images of stable emulsions were taken with an optical microscope (Olympus IX51) with a digital camera (Canon EOS 700D) one day after preparation. A drop of the emulsion cream was placed on a glass slide with a coverslip, unless stated otherwise. Emulsion stability was assessed one day and one month after preparation by measuring the volume of aqueous and organic phases released and the cream height. The fraction of aqueous phase (f_w) or organic phase (f_o) resolved is calculated as in Eq. (S1).

$$f_{o,w} = \frac{h_t}{h_0} \qquad \text{[Eq. (S1)]}$$

where h_t is the height of organic or aqueous phase separated after some time and h_0 is the height of organic or aqueous phase used to prepare the emulsion.

(c) Emulsions with silica particles

Emulsions with ~1 wt.% silica particles of different hydrophilicities (15%, 25%, 51%, 65%, 79% and 100% SiOH) were prepared under the standard procedure. The two phases were: 10 mM *n*-octanaloxime in *n*-dodecane and a 0.44 g L^{-1} *E. coli* cells (containing OxdB) solution in 50 mM K_2HPO_4/KH_2PO_4 buffer (pH = 7). Therefore, the concentration of the substrate and the cells in the emulsion was 0.082 wt.% and 0.025 wt.%, respectively. Two sets of emulsions were prepared by mixing equal volumes of the two phases: one for the study of the long-term emulsion stability and a second one to follow the conversion of the reaction at room temperature at different times. Silica particles of different hydrophilicities were dispersed by

hand-shaking in either the organic (15, 25 and 51% SiOH) or the aqueous (65, 79 and 100% SiOH) phase prior to emulsification.

Emulsions with different concentrations of 65% SiOH silica particles and fixed concentration of *E. coli* cells (containing OxdB) (0.025 wt.%) and substrate (0.082 wt.%) were prepared to determine the influence of particle concentration on both emulsion stability and the conversion of the reaction at room temperature. The particle concentrations studied were: 0.1, 0.2, 0.5, 0.7 and 0.9 wt.%.

(d) Control monophasic system

n-Octanaloxime was placed as a solid in a 0.44 g L^{-1} *E. coli* cells (containing OxdB) solution in 50 mM K_2HPO_4/KH_2PO_4 buffer (pH = 7) and stirred at 1,000 rpm. For each reaction time (1, 4 and 24 h) one individual reaction was performed. The concentration of *n*-octanaloxime in the dispersion was 10 mM.

(e) Control two-phase systems at planar interface

In order to compare the biotransformation in Pickering emulsions with that in the two-phase system, the two phases containing the substrate and the *E. coli* cells (containing OxdB) at the same concentrations as the emulsions prepared previously and at room temperature were put together in a 14 mL screw-cap glass vial. No silica particles were added. The aqueous phase containing *E. coli* cells (containing OxdB) and the organic phase containing *n*-octanaloxime were either added as they are or they were previously homogenised with the Ultra-turrax for 2 min at 13,000 rpm. The two phases were either left to stand or stirred with a magnetic stirrer to enhance the diffusion of the substrate to the interface. The stirring was performed with a 3 x 8 mm magnetic bar and it was kept at 1,000 rpm (IKA RCT classic). Therefore, six two-phase systems were studied containing equal volumes of organic and aqueous phases.

1.2.5 Determination of the conversion of the reaction

(a) Sample preparation for GC

For the Pickering emulsions, 100 μL of the emulsion were added to 400 μL EtOAc. After mixing in a vortexer (VWR analog vortex mini 945304) phase separation was induced by pulse centrifugation to 14,000 g at room temperature (VWR microstar 17). 200 μL of the organic phase was filtered through a small pad of silica (packed in a Pasteur pipette) into a glass vial and analysed by GC (Shimadzu GC 2010 Plus). The conversion of the reaction is defined as

the area of *n*-octanenitrile peak over the total area (*n*-octanenitrile + *n*-octanaloxime). For the determination of conversion after work-up, the Pickering emulsion was broken by centrifugation (5 min at 5,000 rpm) (Thermo Scientific Heraeus multifuge X3R). Both phases were analysed separately. 100 μL of each phase were added to 400 μL EtOAc and after mixing (vortex) phase separation was induced by pulse centrifugation to 14,000 g (VWR microstar 17) at room temperature in the case of the aqueous sample. 200 μL of the organic phase was filtered through a small pad of silica (packed in a Pasteur pipette) into a glass vial and analysed by GC.

For the monophasic system, the whole reaction in the aqueous phase was extracted with 5 mL of EtOAc. 200 μL of the organic phase was transferred to a glass vial for GC analysis.

For the two-phase system (no emulsion), both phases were analysed separately. 100 μL of each phase was added to 400 μL EtOAc, and mixed (vortex) by pulse centrifugation to 14,000 g at room temperature. 200 μL of the organic phase was transferred to a glass vial for GC analysis.

(b) GC method

Conversion of the biotransformation was determined by GC measurements (Shimadzu GC-2010 Plus). The extraction efficiency was proven beforehand by a series of experiments ranging from 1.25 mM to 10 mM. For each concentration three extractions were performed and measured 3 times (see Table S4). Measurements were conducted on a chiral SGE Analytik B6B-174 column (30 m length, 0.25 mm inner diameter, 0.25 μm film thickness) with nitrogen as carrier gas. An injector temperature of 220 °C in a split injection mode (10:1) was used and 1 μL of the sample was injected in one of the following methods.

Method A: The following temperature gradient was used for the *n*-dodecane-containing samples: 130 °C starting temperature (hold 6 min), with 30 °C min^{-1} to 200 °C (hold 2 min). The retention times of *n*-octanenitrile and *n*-octanaloxime are 3.49 and 4.50 min, respectively.

Method B: The following temperature gradient was used for the samples without *n*-dodecane: 140 °C starting temperature (hold 1 min), with 20 °C min^{-1} to 155 °C (hold 3.5 min), with 20 °C min^{-1} to 190 °C (no hold), with 50 °C min^{-1} to 200 °C (no hold). The retention times of *n*-octanenitrile and *n*-octanaloxime are 2.39 and 2.81 min, respectively.

An initial rate was also calculated as shown below by considering that the conversion at t = 0 is 0%.

$$initial\ rate\ (\%\ h^{-1}) = \frac{conversion\ at\ \ t{=}1\ h - conversion\ at\ t_0}{1\ h} \qquad \text{[Eq. (S2)]}$$

1.2.6 Calculation of the total interfacial area in emulsions

If emulsion droplets are considered spherical and monodisperse, then the volume of an emulsion droplet is that of a sphere $4/3\pi r^3$. As the amount of organic phase used to prepare the emulsion was 5 cm^3, by dividing this volume by the volume of one sphere, the number of droplets in each emulsion could be calculated. The total interfacial area in the emulsion is equal to the surface area of one sphere ($4\pi r^2$) times the total number of droplets in the emulsion.

1.2.7 Recyclability study

An emulsion with 1 wt.% fumed silica particles (65% SiOH) was prepared in a 15 mL Falcon tube between an organic phase containing *n*-octanaloxime and an aqueous phase containing *E. coli* cells (with OxdB). The concentration of the substrate and the cells in each phase was the same as in the previous experiments, 10 mM and 0.44 g L^{-1}, respectively. After homogenisation with an Ultra-turrax homogeniser at 13,000 rpm for 2 min, the emulsion was left to stand for 1 hour without stirring. Afterwards, the emulsion was centrifuged for 5 min at 20,000 g and the organic phase was separated with a Pasteur pipette and analysed by GC. The aqueous phase separated containing the cells and silica particles was re-suspended, a fresh volume of *n*-dodecane containing *n*-octanaloxime (10 mM) was added and the two phases were re-homogenised. After 1 h of reaction time, the emulsion was broken by centrifugation and the organic phase analysed. Three cycles were performed in total. The recycling study was performed twice and the average conversion was determined. The average droplet diameter after 1 h was measured for the emulsion prepared in each cycle.

2. Results and Discussion

2.1 Controls to check the surface-activity of the substrate and the product

The surface-activity at the oil-water interface of the substrate (*n*-octanaloxime) and the product (*n*-octanenitrile) of the reaction was evaluated. Dispersions containing either *n*-octanaloxime or *n*-octanenitrile in *n*-dodecane at various concentrations were homogenised with a K_2HPO_4/KH_2PO_4 buffer solution with an Ultra-turrax homogeniser as explained above. After emulsification, photos of the vials were taken at different times to assess the emulsion stability.

As shown in Figure S1, *n*-octanaloxime is not surface-active at the oil-water interface as complete phase separation is attained several min after preparation at all concentrations. The same occurs for emulsions prepared with *n*-octanenitrile (Figure S2). Therefore, these two species are not active at the *n*-dodecane-water interface, as expected from them being small molecules possessing no amphiphilic structure.

2.2 Effect of the concentration of *E. coli* cells on emulsion stability

E. coli is a bacteria, whose outer membrane consists of lipopolysaccharide, phospholipid, protein and Enterobacterial common antigen.[3] Therefore, due to the presence of both hydrophilic and hydrophobic moieties within its structure, it can stabilise emulsions. Here, however, we want silica particles to be the sole emulsifier. Therefore, the concentration below which *E. coli* cells are not capable to stabilise an emulsion has to be identified. In order to do so, a series of emulsions were prepared by increasing the [*E. coli* cells] (from 0.005 to 0.91 wt.%) at constant concentration of *n*-octanaloxime (0.082 wt.%). All the concentrations are given with respect to the final emulsion. Photos of the vials were taken at different times and o/w emulsions were prepared in all cases. As shown in Figure S3 (t = 1 day), at low [*E. coli* cells] emulsions were not stable as the two phases completely phase separate after emulsification. By increasing the [*E. coli* cells], emulsions became more stable and the average droplet diameter decreased (Figure S4 and Figure S5(a)). The stability of these emulsions did not change substantially after 1 month. The fraction of aqueous and organic phase released was measured one month after preparation (Figure S5(b)). Coalescence is not fully inhibited at any of the concentrations investigated despite the fraction of oil resolved decreasing by increasing the concentration of cells. Creaming was not inhibited in any case, with the minimum fraction of aqueous phase resolved being as high as 0.7. Based on these results, the concentration of *E. coli* cells selected for the preparation of emulsions with silica particles was set to 0.025 wt.%.

E. coli cells do not contain the Oxd cells. However, the *E. coli* cells containing OxdB should have the same surface properties as the empty *E. coli* cells. An emulsion without silica particles containing an organic phase comprising of *n*-octanaloxime in *n*-dodecane and an aqueous solution containing the *E. coli* cells (containing OxdB) in 50 mM K_2HPO_4/KH_2PO_4 buffer (pH = 7) was prepared. The concentrations of *n*-octanaloxime and *E. coli* cells (containing OxdB) in the emulsion were 0.082 wt.% and 0.025 wt.%, respectively. As shown in Figure S6, complete phase separation was achieved several seconds after homogenisation, showing the same behaviour as the emulsion prepared with empty *E. coli* cells.

2.3 Limited coalescence model of particle-stabilised emulsions

In particle-stabilised emulsions in which the initial emulsifier concentration is varied, two main régimes can be distinguished with respect to emulsion formation.[4] At low stabilizer concentration (emulsifier-poor régime), droplet interfaces are partially covered by the particulate emulsifier. As a result, droplets coalesce to a limited extent once homogenisation is halted. By increasing the particle concentration, the degree of interfacial coverage increases and the average droplet diameter decreases. This leads to an increase of the total interfacial area between oil and water and prevents further coalescence events.[5] However, at high emulsifier concentrations (emulsifier-rich régime) the interfaces are sufficiently covered by particles and the average droplet diameter does not decrease further by increasing the particle concentration. Therefore, the amount of particles available determines the final droplet surface area, as well as their packing at interfaces. Assuming all particles are spherical and become adsorbed at the interface, the surface coverage C can be defined as the ratio of the interfacial area S_a that can be covered by particles of diameter d_p and the total interfacial area, S_c, equal to $6V_d/D$ where V_d is the volume of disperse phase and D is the drop diameter.[6] For a hexagonal close-packed monolayer of monodisperse particles, C should be equal to around 0.9. If S_a is,

$$S_a = \frac{3m_p}{2\rho_p d_p} \qquad \text{[Eq. (S3)]}$$

where ρ_p is the particle density (g cm^{-3}). Then,

$$\frac{1}{D} = \frac{m_p}{4CV_d\rho_p d_p} \qquad \text{[Eq. (S4)]}$$

By plotting the inverse of the droplet diameter *versus* the mass of the particles, m_p a straight line is expected to be obtained. From the slope, and considering that ρ_p is 0.18 g cm^{-3} and d_p is 20 nm (primary particle diameter),[2] C can be determined. The surface coverage calculated is 22. As it is > 0.9, this suggest that particles are closely packed at the interface and there is more than one layer of particles and some excess particles in the continuous aqueous phase. For this calculation, the primary particle diameter has been accounted as the particle diameter. However, particle aggregates instead of discrete particles are more likely to be present at the interface, so a lower value of surface coverage is more reasonable.

3. References

[1] H. Balard, E. Papirer, A. Khalfi and H. Barthel, *Compos. Interfaces*, **1999**, *6*, 19.

[2] B.P. Binks, S.O. Lumsdon, *Langmuir* **2000**, *16*, 8622–8631.

[3] B. Lugtenberg, *Trends Biochem. Sci.* **1981**, *6*, 262–266

[4] S. Tcholakova, N.D. Denkov, A. Lips, *Phys. Chem. Chem. Phys.* **2008**, *10*, 1608–1627.

[5] S. Arditty, C.P. Whitby, B.P. Binks, V. Schmitt, F. Leal-Calderon, *Eur. Phys. J. E* **2003**, *11*, 273–281.

[6] B.P. Binks, S.O. Olusanya, *Chem. Sci.* **2017**, *8*, 708–723.

4. Appendix

Oxd Sequences

Oxd from *Bacillus* sp. OxB-1 (OxdB)
(Accession number: GenBank: AP013294.1)

Base sequence (codon-optimized for *E. coli*):

ATGAAAAATATGCCGGAAAATCACAATCCACAAGCGAATGCCTGGACTGCCGAATTTCC
TCCTGAAATGAGCTATGTAGTATTTGCGCAGATTGGGATTCAAAGCAAGTCTTTGGATCA
CGCAGCGGAACATTTGGGAATGATGAAAAAGAGTTTCGATTTGCGGACAGGCCCCAAAC
ATGTGGATCGAGCCTTGCATCAAGGAGCCGATGGATACCAAGATTCCATCTTTTTAGCCT
ACTGGGATGAGCCTGAAACATTTAAATCATGGGTTGCGGATCCTGAAGTACAAAAGTGG
TGGTCGGGTAAAAAAATCGATGAAAATAGTCCAATCGGGTATTGGAGTGAGGTAACGAC
CATTCCGATTGATCACTTTGAGACTCTTCATTCCGGAGAAAATTACGATAATGGGGTTTC
ACACTTTGTACCGATCAAGCATACAGAAGTCCATGAATATTGGGGAGCAATGCGCGACC
GCATGCCGGTGTCTGCCAGTAGTGATTTGGAAAGCCCCCTTGGCCTTCAATTACCGGAAC
CCATTGTCCGGGAGTCTTTCGGAAAACGGCTAAAAGTCACGGCGCCGGATAATATTTGCT
TGATTCGAACCGCTCAAAATTGGTCTAAATGTGGTAGCGGGGAAAGGGAAACGTATATA
GGACTAGTGGAACCGACCCTCATAAAAGCGAATACGTTTCTTCGTGAAAATGCTAGTGA
AACAGGCTGTATTAGTTCAAAATTAGTCTATGAACAGACCCATGACGGCGAAATAGTAG
ATAAATCATGTGTCATCGGATATTATCTCTCCATGGGGCATCTTGAACGCTGGACGCATG
ATCATCCAACACATAAAGCGATCTACGGAACCTTTTATGAGATGTTGAAAAGGCATGATT
TTAAGACCGAACTTGCTTTATGGCACGAGGTTTCGGTGCTTCAATCCAAAGATATCGAGC
TTATCTATGTCAACTGCCATCCGAGTACTGGATTTCTTCCATTCTTTGAAGTGACAGAAAT
TCAAGAGCCTTTACTGAAAAGCCCTAGCGTCAGGATCCAGTGA

Amino acid sequence:

MKNMPENHNPQANAWTAEFPPEMSYVVFAQIGIQSKSLDHAAEHLGMMKKSFDLRTGPKH
VDRALHQGADGYQDSIFLAYWDEPETFKSWVADPEVQKWWSGKKIDENSPIGYWSEVTTIPI
DHFETLHSGENYDNGVSHFVPIKHTEVHEYWGAMRDRMPVSASSDLESPLGLQLPEPIVRES
FGKRLKVTAPDNICLIRTAQNWSKCGSGERETYIGLVEPTLIKANTFLRENASETGCISSKLVY
EQTHDGEIVDKSCVIGYYLSMGHLERWTHDHPTHKAIYGTFYEMLKRHDFKTELALWHEVS
VLQSKDIELIYVNCHPSTGFLPFFEVTEIQEPLLKSPSVRIQ

^{1}H and ^{13}C-NMR spectra of *n*-octanaloxime

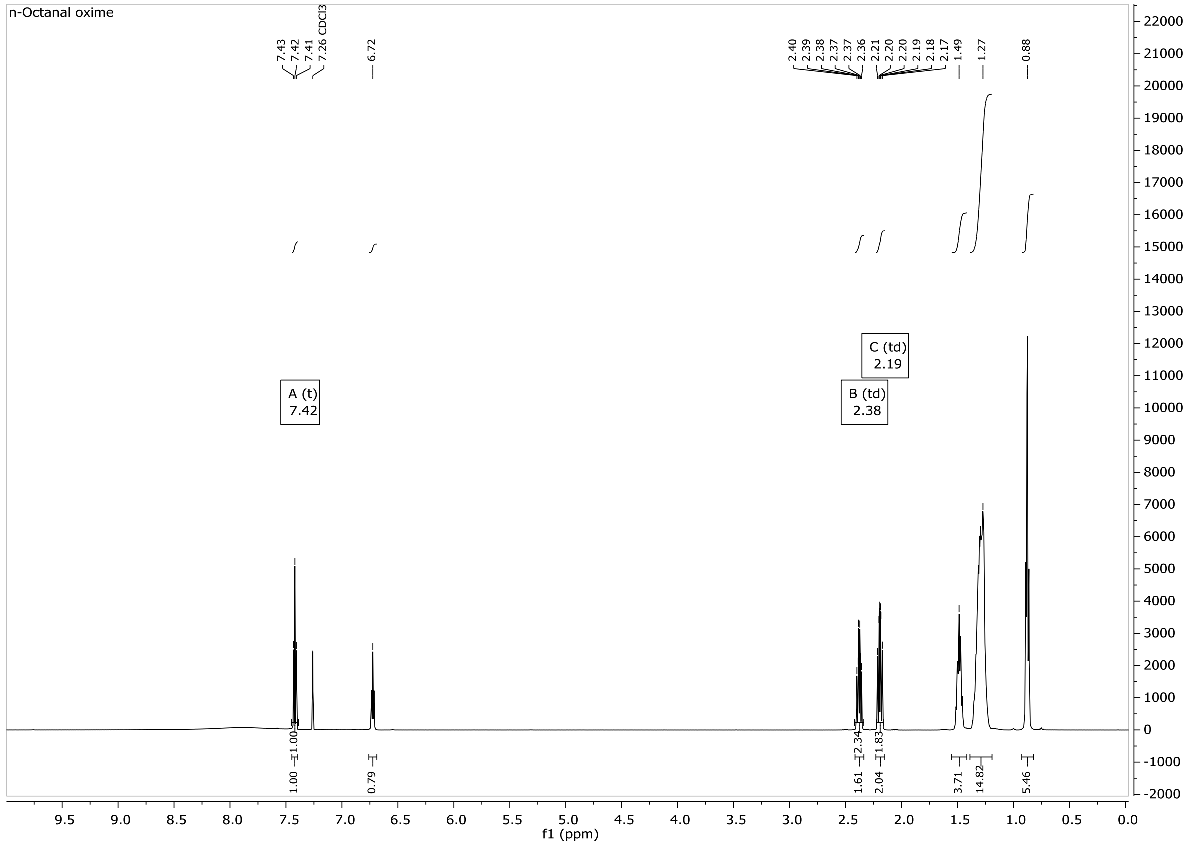

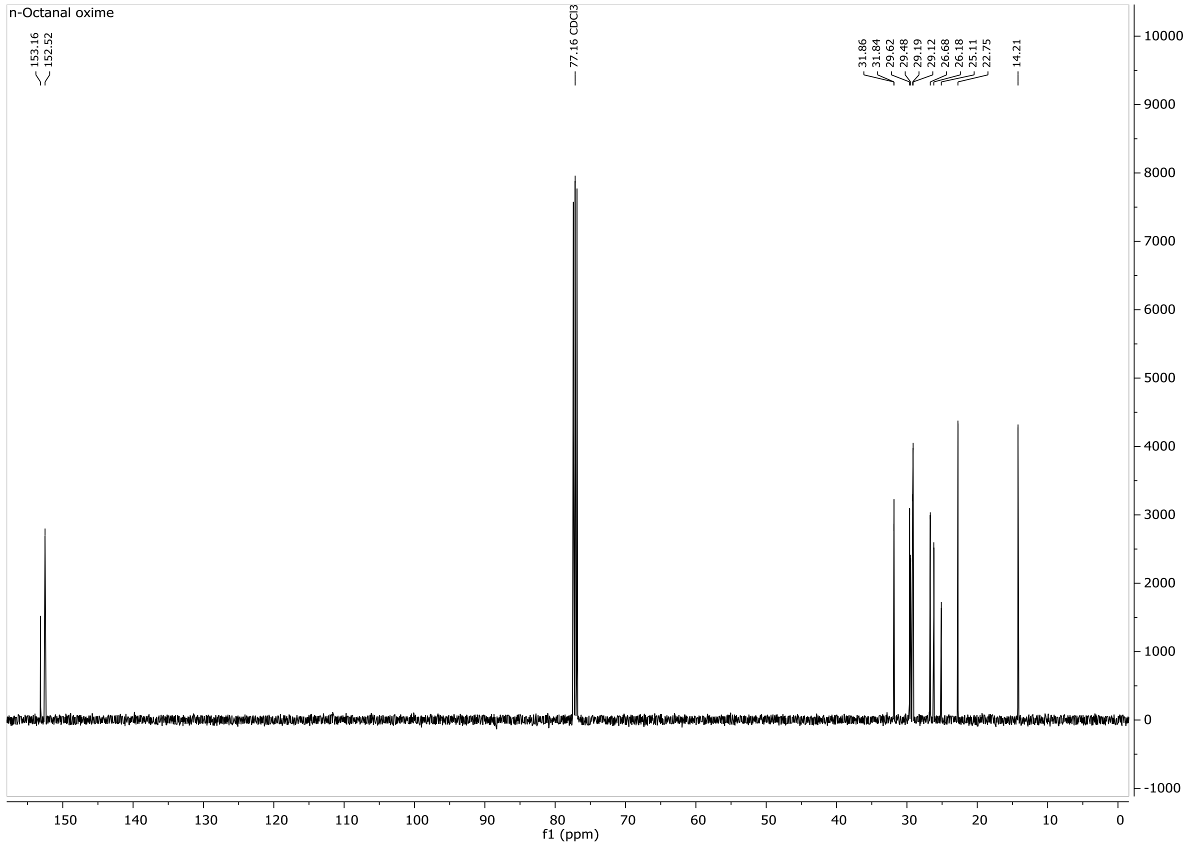

Table S1. Origin of the Oxd-genes, used vector constructs, provider of the vector constructs and marker-resistance of the constructs.

Origin of Oxd-gene	Vector construct	Oxd	Provider	Resistance
Bacillus sp. OxB-1	pUC18_OxdB	OxdB	Asano group	Carbenicillin

Table S2. Conversion of C_8-aldoximes to the related nitrile determined by GC. Activity test performed according to experimental procedure, taken from planar interface.

#	Sample no.	Cell amount (bww) /g L^{-1}	[*n*-octanaloxime]/mM	Conversion (GC) /%
**1**	1	33	100	45
**2**	2	33	100	44
**3**	3	33	100	42
**4**	**Average**	**33**	**100**	**44**
**5**	1	0.44	10	62
**6**	2	0.44	10	63
**7**	3	0.44	10	62
**8**	**Average**	**0.44**	**10**	**62**

Table S3. Concentration of *E. coli* cells given in the aqueous dispersion and in the final emulsion.

[*E. coli* cells]$_{\text{aqueous dispersion}}$/g L^{-1}	[*E. coli* cells]$_{\text{emulsion}}$/wt.%
15.92	0.91
8.75	0.5
4.37	0.25
1.75	0.1
1.31	0.075
0.87	0.050
0.44	0.025
0.17	0.01
0.09	0.005

Table S4. Extraction analysis of C_8-oxime and C_8 nitrile taken from planar interface.

Concentration	Extraction no.	% C_8-nitrile	% C_8-oxime
**10 mM**	stock	63.2	36.8
	1	63.0	37.0
	2	66.5	33.5
	3	62.9	37.1
	**Average (extractions)**	**64.2**	**35.8**
**5 mM**	stock	69.0	31.0
	1	69.9	30.1
	2	65.9	34.1
	3	66.0	34.0
	**Average (extractions)**	**67.3**	**32.7**
**2.5 mM**	stock	74.5	25.5
	1	71.6	28.4
	2	74.2	25.8
	3	74.8	25.2
	**Average (extractions)**	**73.5**	**26.5**
**1.25 mM**	stock	84.9	15.1
	1	77.1	22.9
	2	77.4	22.6
	3	77.5	22.5
	**Average (extractions)**	**77.3**	**22.7**

Table S5. Average droplet diameter of the emulsions measured 1 h after preparation and conversion of the reaction and relative activity measured from the organic phase separated after three cycles for two independent runs. The initial emulsion (1) is prepared by mixing equal volumes of an aqueous phase containing *E. coli* cells (0.44 g L^{-1}) in 50 mM K_2HPO_4/KH_2PO_4 buffer (pH = 7) and an organic phase containing *n*-octanaloxime dispersed in *n*-dodecane (10 mM). 65% SiOH silica particles (1 wt.% in the emulsion) were used as the sole emulsifier. The subsequent emulsions (2 and 3) were prepared with the aqueous phase separated after centrifugation (containing the cells and the silica particles) and an equal volume of a fresh organic phase containing *n*-octanaloxime (10 mM).

**Cycle number**	**Average droplet diameter/μm**	**Conversion/%**			**Relative activity/%**
		**Rep. 1**	**Rep. 2**	**Average**	
**1**	37.2 ± 10.5	6.5	6.9	6.7	100
**2**	49.9 ± 15.3	6.8	6.5	6.7	99.3
**3**	62.0 ± 19.2	6.5	6.0	6.3	93.3

Figure S1. Appearance of emulsions at different times (given) prepared with equal volumes of a 50 mM K_2HPO_4/KH_2PO_4 buffer (pH = 7) and an organic phase containing *n*-octanaloxime in *n*-dodecane. Homogenisation was carried out with an Ultra-turrax homogeniser at 13,000 rpm for 2 min. [*n*-octanaloxime] in the organic phase is: (a) 0.01 M, (b) 0.05 M, (c) 0.1 M and (d) 0.5 M. Scale bars = 1 cm.

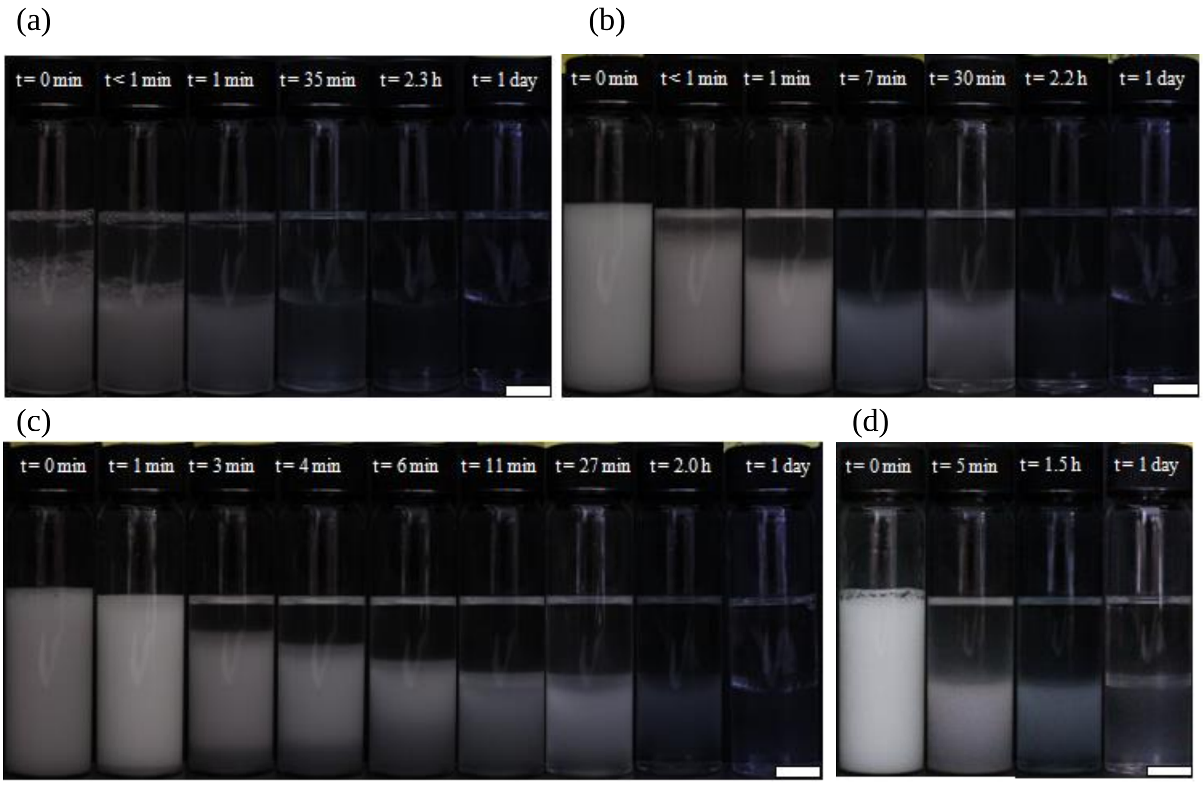

Figure S2. Appearance of emulsions at different times (given) prepared with equal volumes of a 50 mM K_2HPO_4/KH_2PO_4 buffer (pH = 7) and an organic phase containing *n*-octanenitrile in *n*-dodecane. Homogenisation was carried out with an Ultra-turrax homogeniser at 13,000 rpm for 2 min. [*n*-octanenitrile] in the organic phase is: (a) 0.01 M, (b) 0.05 M, (c) 0.5 M and (d) 1 M. Scale bars = 1 cm.

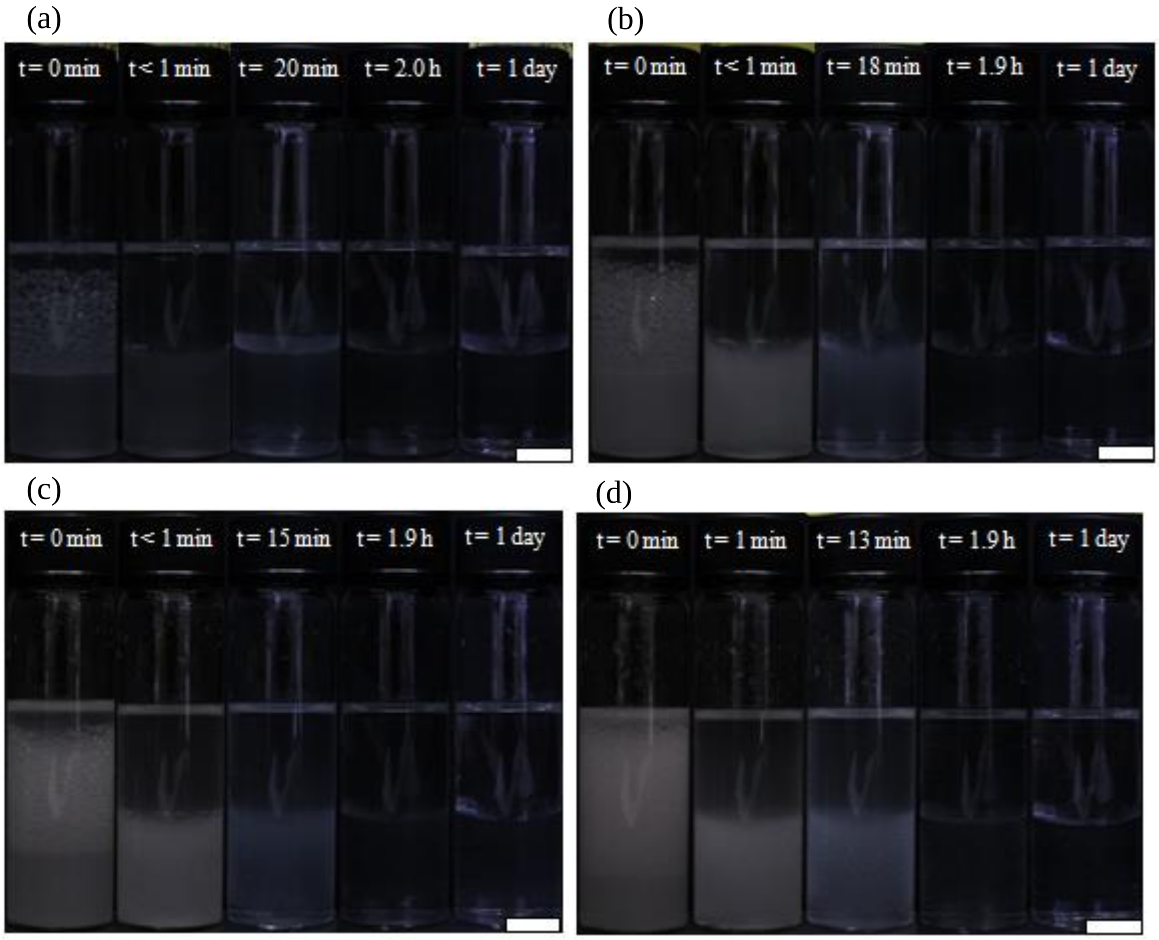

Figure S3. Appearance of emulsions prepared at different [*E. coli* cells]$_{emulsion}$ (given) (a) 1 day and (b) 1 month after preparation. Each emulsion is prepared by mixing equal volumes of an aqueous phase containing *E. coli* cells in 50 mM K_2HPO_4/KH_2PO_4 buffer (pH = 7) and an organic phase containing *n*-octanaloxime dispersed in *n*-dodecane (0.082 wt.% in the emulsion, 10 mM in the organic phase). Scale bars = 1 cm.

(a)

(b)

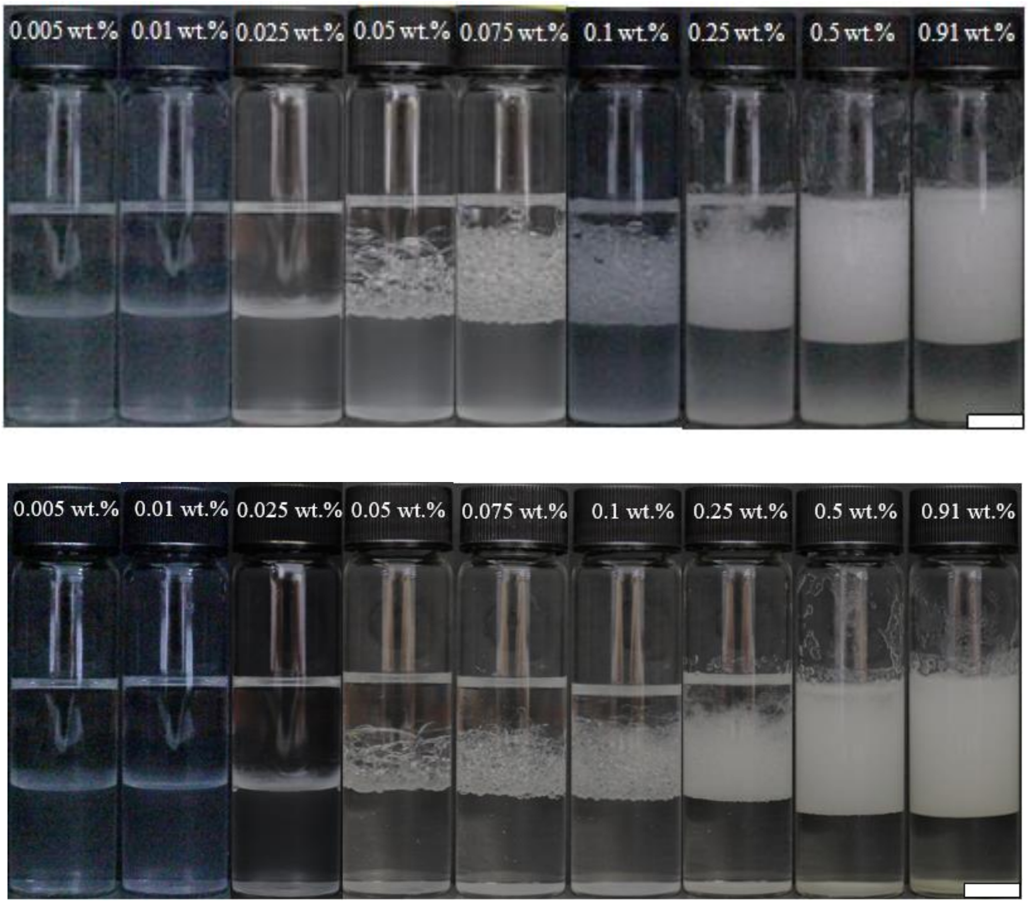

Figure S4. Optical microscope images of o/w emulsions in Figure S3(a) at different [*E. coli* cells]$_{\text{emulsion}}$ (given). Images taken 1 day after preparation.

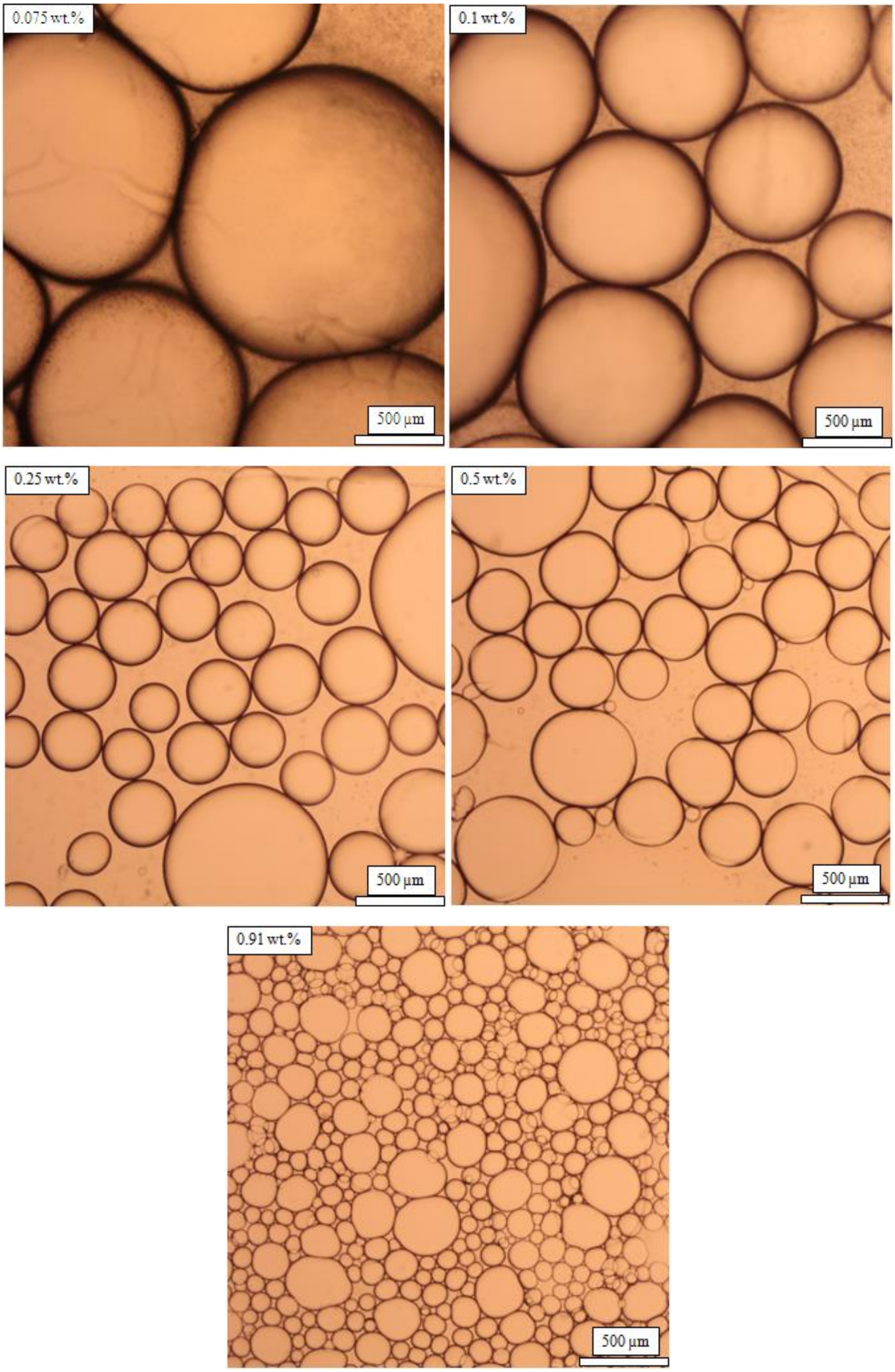

Figure S5. Plot of (a) average droplet diameter 1 day and 1 month after preparation and (b) fraction of organic and aqueous phase resolved after 1 month *versus* [*E. coli* cells]$_{emulsion}$ in the o/w emulsions in Figure S3.

(a)

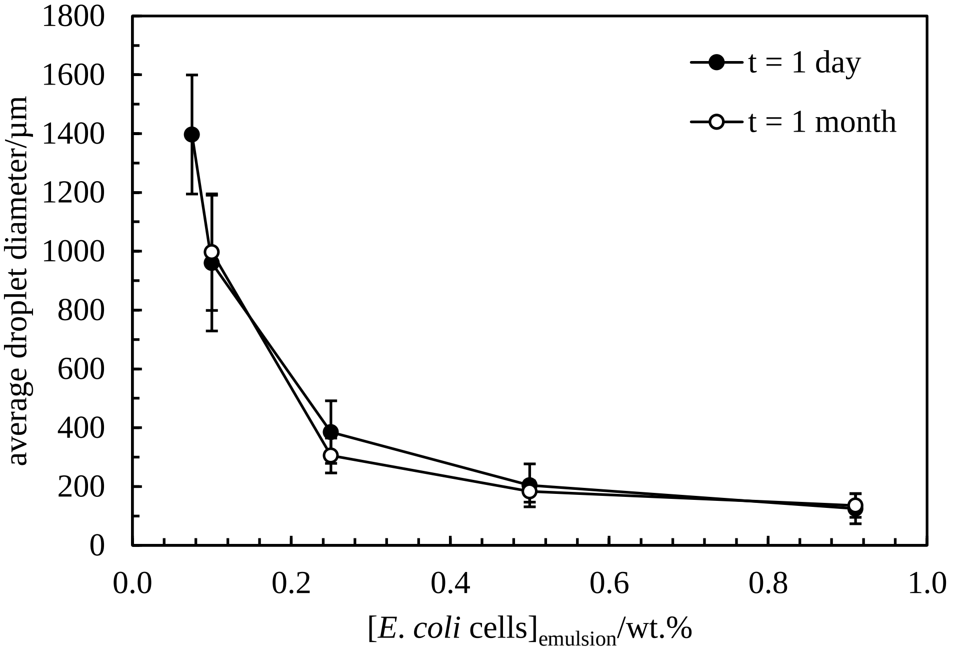

(b)

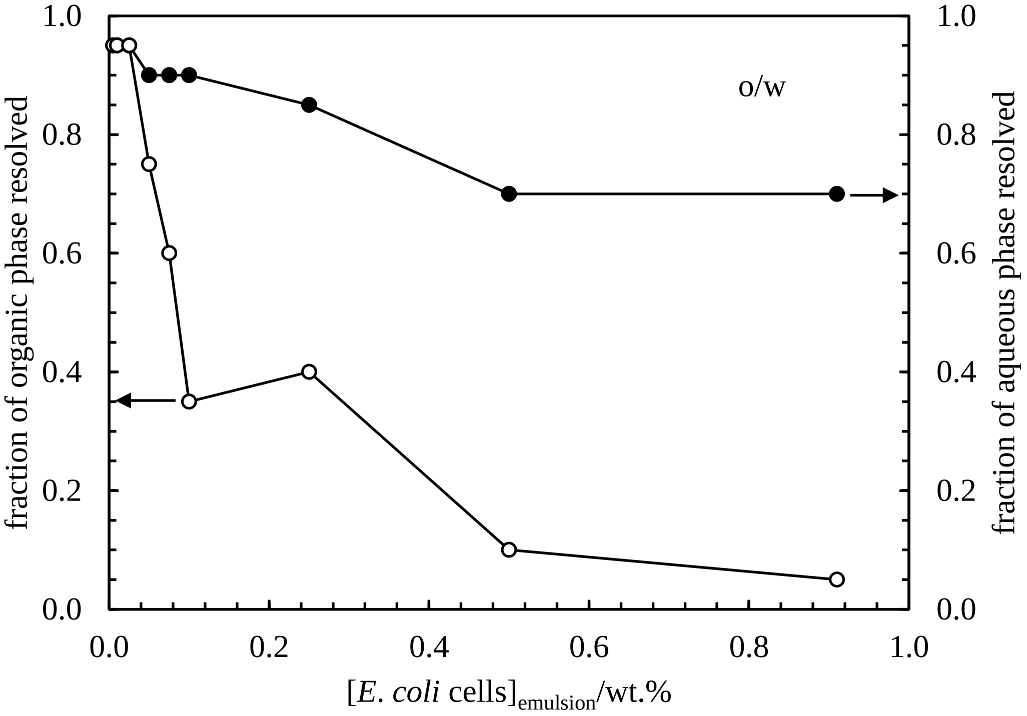

Figure S6. Appearance of an emulsion at different times (given) prepared after mixing equal volumes of an aqueous phase containing *E. coli* cells (containing OxdB) in 50 mM K_2HPO_4/KH_2PO_4 buffer (pH = 7) (0.025 wt.% in the emulsion) and an organic phase containing *n*-octanaloxime dispersed in *n*-dodecane (0.082 wt.% in the emulsion, 10 mM in the organic phase). Scale bar = 1 cm.

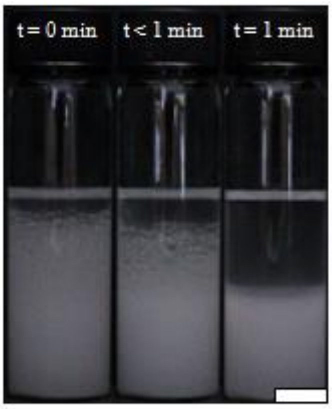

Figure S7. Optical microscope images of emulsions in Figure 1 prepared with silica particles of various % SiOH. Images taken 1 day after preparation.

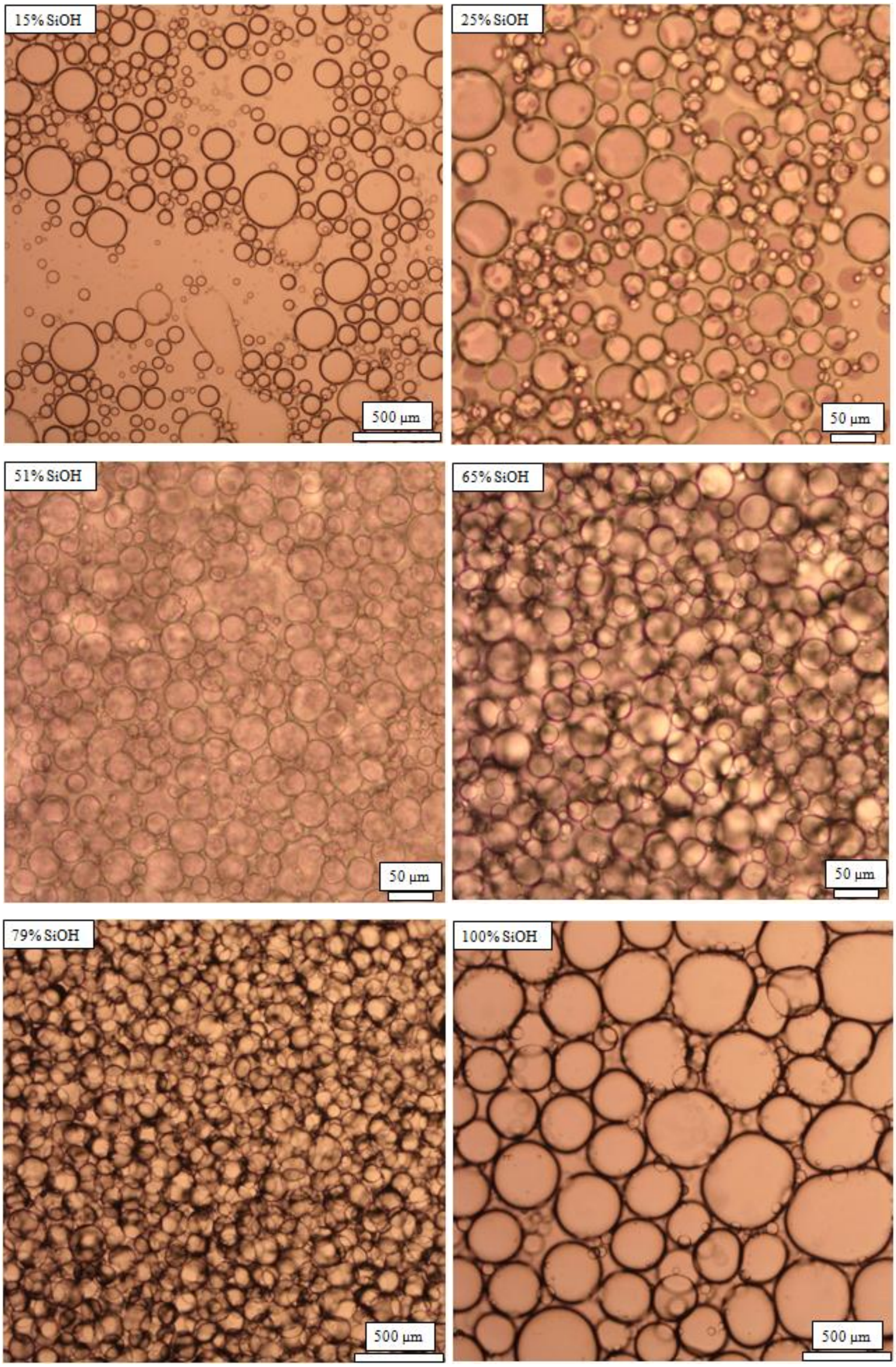

Figure S8. Plot of the conversion of the reaction measured in the organic phase *versus* time for the two-phase control systems. (a) The aqueous phase in two of the controls was previously homogenised with an Ultra-turrax under the same conditions as for the emulsion preparation (13,000 rpm for 2 min) (hom.). In the other two controls, the aqueous phase was not homogenised (non-hom.). The two-phase systems were either stirred (stirr.) or not stirred (non-stirr.) throughout the time. (b) The aqueous and the organic phases (without silica particles) were homogenised together with an Ultra-turrax (13,000 rpm for 2 min). Afterwards, the two phases were left either stirred or non-stirred throughout the time. The organic phase in all cases consists of a 10 mM dispersion of *n*-octanaloxime in *n*-dodecane and the aqueous phase is a 0.44 g L^{-1} solution of *E. coli* cells in 50 mM K_2HPO_4/KH_2PO_4 buffer (pH = 7).

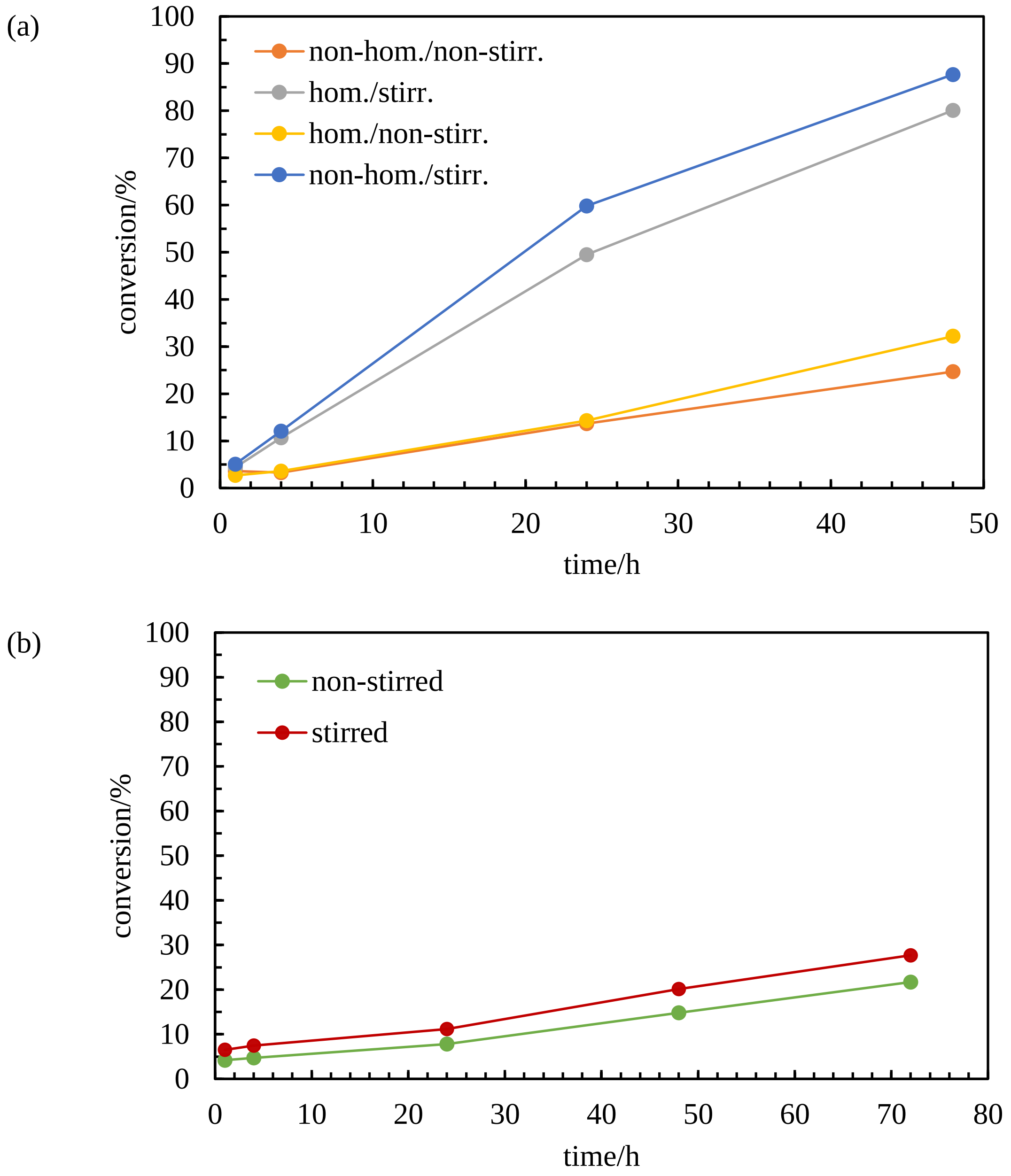

Figure S9. (a) Schematic representation of water-in-oil Pickering emulsion droplets stabilised by silica particles of two different hydrophobicities. The contact angle is measured through the water phase. (b) Plot of the conversion of the reaction *versus* %SiOH for the emulsions in Figure 1 after 48 h of reaction time and conversion measured in the organic phase recovered after breaking the emulsion stability by centrifugation at t = 48 h (WU OP). The horizontal dashed line indicates the conversion after 48 h of a control containing a 0.44 g L^{-1} *E. coli* cells (containing OxdB) dispersion in 50 mM K_2HPO_4/KH_2PO_4 buffer (pH = 7) and a 10 mM *n*-octanaloxime solution in *n*-dodecane. In the control, the aqueous dispersion was previously homogenised with an Ultra-turrax (13,000 rpm for 2 min) and the two-phase system was left to stand for 48 h without stirring.

(a)

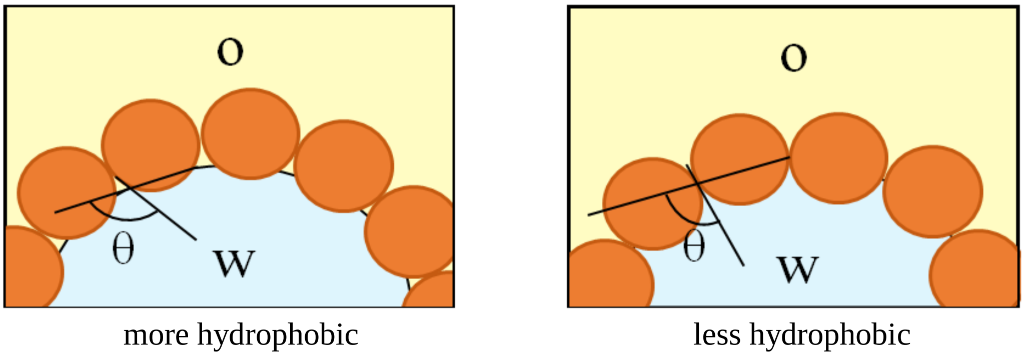

(b)

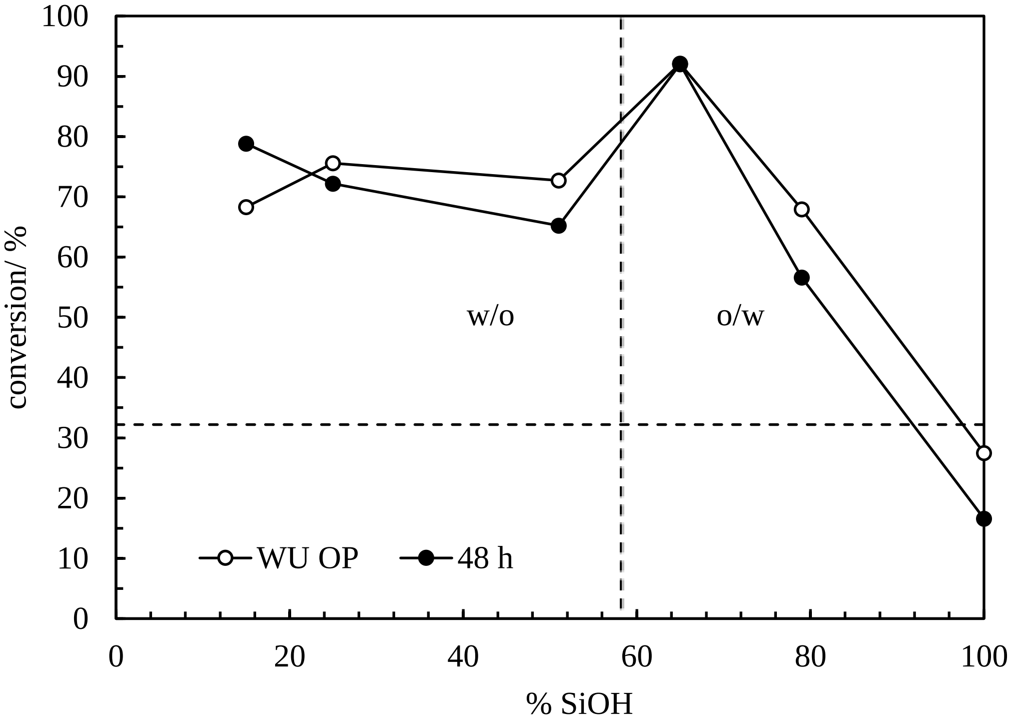

Figure S10. Optical microscope images of o/w emulsions in Figure 4(a) 1 day after preparation at different magnifications. Images taken on a glass slide without coverslip.

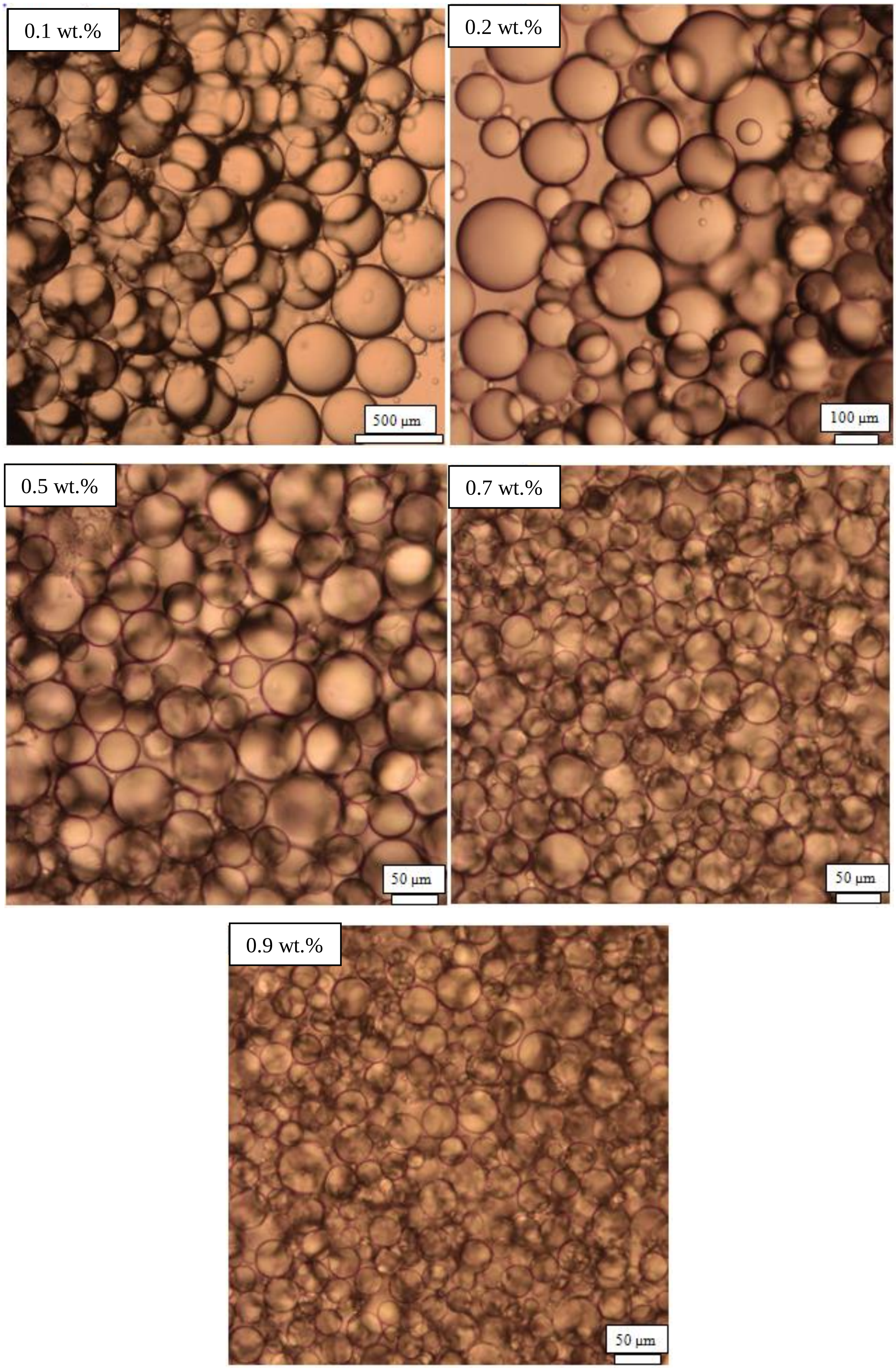

Figure S11. Plot of the conversion of the reaction *versus* concentration of 65% SiOH silica particles for the o/w emulsions in Figure 4(a) after 48 h and conversion measured in organic phase recovered after centrifugation of the emulsions at t = 48 h (WU OP). The dashed line indicates the conversion after 48 h of a control containing a 0.44 g L^{-1} *E. coli* cells (containing OxdB) dispersion in 50 mM K_2HPO_4/KH_2PO_4 buffer (pH = 7) and a 10 mM *n*-octanaloxime solution in *n*-dodecane. In the control, the aqueous dispersion was previously homogenised with an Ultra-turrax (13,000 rpm for 2 min) and the two-phase system was left to stand for 48 h without stirring.

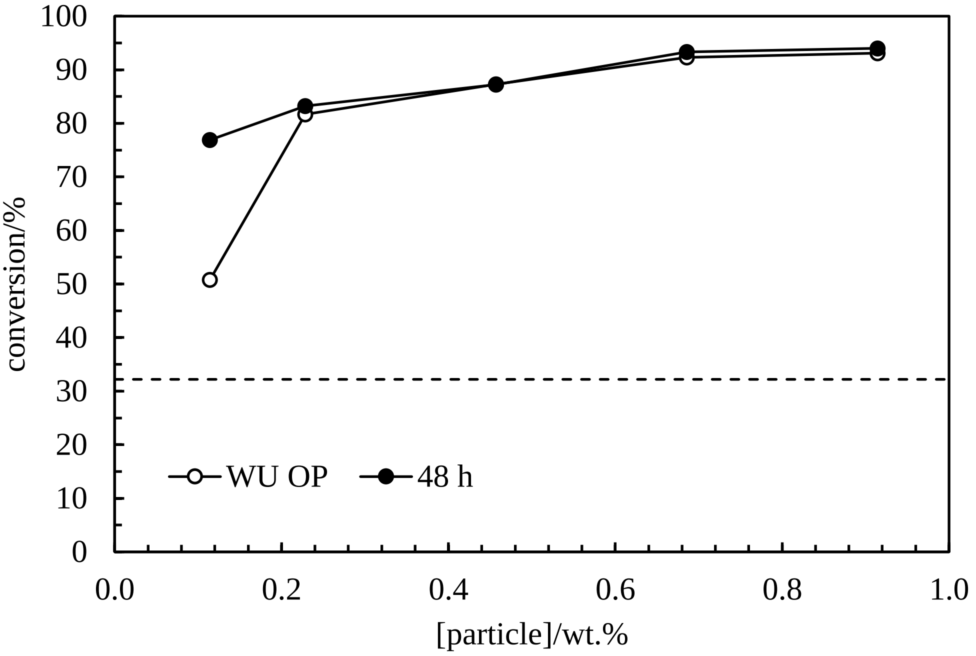

Supplement: Supplementary file 1 — Supplementary [file ANIE-60-1450-s001.pdf]
